# Novel Evidence of HBV Recombination in Family Cluster Infections in Western China

**DOI:** 10.1371/journal.pone.0038241

**Published:** 2012-06-04

**Authors:** Bin Zhou, Zhanhui Wang, Jie Yang, Jian Sun, Hua Li, Yasuhito Tanaka, Masashi Mizokami, Jinlin Hou

**Affiliations:** 1 Institute of Hepatology, Nanfang Hospital, Southern Medical University, Guangzhou, Guangdong, China; 2 Qinghai Provincial Infectious Diseases Hospital, Xining, Qinghai, China; 3 Department of Virology and Liver Unit, Nagoya City University Graduate School of Medical Sciences, Kawasumi, Mizuho, Nagoya, Japan; 4 The Research Center for Hepatitis and Immunology, National Center for Global Health and Medicine, Kounodai, Ichikawa, Japan; Institute of Infectious Disease and Molecular Medicine, South Africa

## Abstract

Two hepatitis B virus (HBV) C/D recombinants were isolated from western China. No direct evidence indicates that these new viruses arose as a result of recombination between genotype C and D or a result of convergence. In this study, we search for evidence of intra-individual recombination in the family cluster cases with co-circulation of genotype C, D and C/D recombinants. We studied 68 individuals from 15 families with HBV infections in 2006, identified individuals with mixed HBV genotype co-infections by restriction fragment length polymorphism and proceeded with cloning and DNA sequencing. Recombination signals were detected by RDP3 software and confirmed by split phylogenetic trees. Families with mixed HBV genotype co-infections were resampled in 2007. Three of 15 families had individuals with different HBV genotype co-infections in 2006. One individual (Y2) had a triple infection of HBV genotype C, D and C/D recombinant in 2006, but only genotype D in 2007. Further clonal analysis of this patient indicated that the C/D recombinant was not identical to previously isolated CD1 or CD2, but many novel recombinants with C2, D1 and CD1 were simultaneously found. All parental strains could recombine with each other to form new recombinant in this patient. This indicates that the detectable mixed infection and recombination have a limited time window. Also, as the recombinant nature of HBV precludes the possibility of a simple phylogenetic taxonomy, a new standard may be required for classifying HBV sequences.

## Introduction

Not all viruses are equally prone to recombination. Recombination has not been detected in several viruses despite repeated searches [1]. Whether recombination does or does not exist is important for understanding the evolution and replication mechanism of a specific kind of virus. Hepatitis B virus (HBV), a major human pathogen, has been classified into 10 genotypes and several sub-genotypes [2], [3]. Many sub-genotypes were identified by polygenetic analysis as recombinants. But there is no direct evidence to indicate that these subgenotypes arose as a result of recombination or perhaps a result of convergence.

Coinfection with different HBV genotype strains is a prerequisite for recombination. As more than one genotype is predominant in most of the geographic regions, coinfection between the predominating HBV genotypes is not a rare finding, especially for B and C, or A and D. The prevalence of mixed HBV genotype infections has been reported using varied genotyping methods [4], [5], [6].

Our previous study found two kinds of HBV C/D recombinants in northwest China [7]. In a further study of ethnic groups of five provinces, we confirmed the geographic and ethnic distribution of the HBV C/D recombinant in northwest China [8], and found that family-cluster HBV infections were common in these endemic areas. We hypothesize that infected members of HBV family clusters would gain exposure to various genotypes through marriage, while at the same time; competent strains would be selected through vertical transmission. It would be useful to observe the mixed infection in family-cluster cases, especially in patients infected with C/D recombinants.

The aim of this study was to evaluate the possibility of recombination between two HBV genotypes within an individual by finding cluster-infected families in which individual members were infected with different HBV genotypes. We would then look for individuals within these families with multiple-genotypes that were likely to have been obtained from other family members as a result of vertical or horizontal transmission. Novel viral genomes within an individual with a multiple genotype infection that were mosaics of the known viral genotypes in the family, but not present in any of the other family members, would be consistent with the hypothesis that they arose within the individual with multiple genotype infections.

## Methods

### Subjects

We enrolled 68 patients with a chronic HBV infection from 15 families. All the families were from a district located at the boundary of Gansu and Qinghai provinces, where the prevalence of genotype C2, D1 and C/D recombinant HBV were known to be high [8]. The families were initially identified with cluster HBV infection in an epidemiological survey in 2002. Sixty-eight individuals were sampled in June 2006 and December 2007 for the purpose of assigning HBV genotypes to chronically infected individuals and finding individuals with multiple HBV genotype co-infections. None of the patients received anti-viral therapy or immunosuppressant drugs. A written, informed consent was obtained from each family, and the study protocol was approved by the Southern Medical University Ethics Committee.

### HBV DNA Extraction and HBV Genotyping

HBV DNA was extracted from 400 µL of serum by QIAamp UltraSens Virus Kit (Qiagen GmbH, Germany), then re-suspended in 50 µL water and stored at −20°C until analysis. HBV genotypes, including C/D recombinant, were initially assigned using the PCR based restriction fragment length polymorphism (RFLP) methods described previously [9], [8].

### Cloning of Mixed Infection Samples

For samples with mixed genotype infections, PCR cover HBV S gene (nt136-1110) was performed using the primers and thermocycling conditions descirbed by Sugauchi et al [10]. For samples needing further recombination analysis, PCR was performed using the primers and thermocycling conditions described by Günther to obtain full-length HBV genome [11]. Alternatively, a nested PCR was used to produce two overlapping fragments in subjects with low HBV DNA levels as described by Sugauchi et al [12]. The spanning of fragment A cover nucleotides 2813 to 1824, and fragment B included nucleotides 1821 to 237. LA-Taq (TAKARA, Japan) and high-fidelity polymerase COD-FX (TOYOBO, Japan) were used to produce amplimers for cloning and direct sequencing respectively. Finally, Fragment C (HBV nt56-nt1824) was obtained from a PCR amplification of Y2 HBV-DNA to which an aliquot of genotype B HBV-DNA had been added. The purpose of this experiment with in-tube control of genotype B was to determine if the recombinant clones were being generated during the PCR amplification. PCR products were gel-purified and cloned into the PMD19-T vector (TAKARA, Japan) according to the manufacturer’s instructions, and used to transform JM109 competent cells (TAKARA, Japan). A minimum of 15 clones were sequenced from subjects with a mixed-strain infection and three clones were sequenced from family members with a single-strain infection. All sequencing of clones and PCR products was performed by Invitrogen Ltd. (Shanghai, China).

### Phylogenetic and Recombination Analysis

Genotypes of clones were determined by phylogenetic tree analysis and recombination analysis. The sequences were assembled using SeqMan II software (DNAStar Inc.). Sequence alignments were performed using ClustalW and confirmed by visual inspection. Phylogenetic trees were constructed by the neighbour-joining (NJ) method (Saitou & Nei, 1987). To confirm the reliability of the phylogenetic tree analysis, bootstrap resampling and reconstruction were carried out 1000 times. A phylogenetic tree analysis̀ of HBV strains isolated from the mixed infection family was compared with reference strains from GenBank. Accession numbers are indicated on the tree. Bootstrap values are shown along each main branch. The lengths of the horizontal bars indicate the number of nucleotide substitutions per site. The regions included in the analysis were the same with fragment A, B and C or a little shorter. Phylogenetic and molecular evolutionary analyses were conducted using MEGA version 5 (Tamura, Peterson, Stecher, Nei, and Kumar 2011).

Recombination signals were initially detected by RDP3.β.4 software [13], [14]. Bootscan, Geneconv and Siscan were used. The highest acceptable P-value was 0.05. Bootscan and Siscan window sizes were 300 bp, step size was 30, replicates for 100 times. A genotype F sequence (GenBank accession numbers is X75658 and X75663) was used as external reference. The precise map of recombination was determined by split phylogenetic tree and alignment. Split phylogenetic trees were constructed by the method same as above. In alignment, each clone was compared to reference C2, D1 and CD1 consensus sequences. We then inspected the alignments to determine the identical crossover sequences around the breakpoint within which the recombination occurred.

### Accession Number of the Sequences

GenBank accession number of reference sequences of HBV genotype C2, D1, CD1 and CD2 are indicated in phylogenetic tree. Accession Numbers of Y2 clones are JX036326-JX036359.

## Results

### Mixed-genotype Infections in HBV Cluster Families

**Figure 1:**
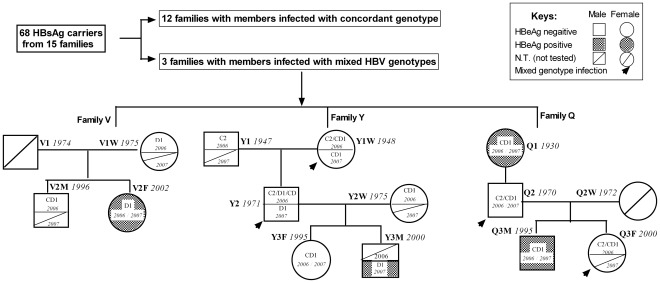
Flow of participants in the study and family trees of family with mixed genotypes/subgenotypes HBV infection. Circles and rectangles correspond to female and male individuals, respectively. Family name and birth date of the patients are indicated beside the circles and rectangles. Subgenotype and the year of blood sampling are indicated inside the circles and rectangles. Family V with affected members across two generations and two genotypes/subgenotypes. Family Q with affected members across three generations and two genotypes/subgenotypes. Family Y with affected members across three generations and three genotypes/subgenotypes. Specially, father (Y2) of family Y with triplicate infection of genotype C, D and CD recombinant in 2006. N.T: Not tested for HBV DNA level below the detection limit of the nested PCR assay or no serum was available.

Different HBV genotypes were found in three families among 15 families. The flow of participants in the study and family trees of families with mixed genotypes/subgenotypes of HBV infection are shown (Figure 1).

Family V had infected members across two generations and two genotypes: In 2006, the mother (V1W) and daughter (V2F) were infected with subgenotype D1 while the son (V2M) had a CD1 recombinant. In 2007, the daughter (V2F) had subgenotype D1 while other family members had HBV DNA levels below the detection limit of the nested PCR assay.

Family Q had infected members across three generations and two genotypes/subgenotypes. In 2006, the grandmother (Q1W) and grandson (Q3M) were infected with CD1 recombinant while father (Q2) and granddaughter (Q3F) had mixed infections of genotype C2 and CD1 recombinants. In 2007, the same genotypes were detected in all family members except that the granddaughter (Q3F) had an HBV DNA level below the detection limit of the nested PCR assay.

Family Y had affected members across three generations and three genotypes/subgenotypes. In 2006, the grandfather of family Y (Y1) was infected with genotype C2 while grandmother (Y1W) had mixed infections of CD1 and C2. Mother (Y2W) and granddaughter (Y3F) were infected with the CD1 recombinant. Father (Y2) had triplicate infections of genotype C2, D1 and CD recombinant. Grandson’s (Y3M) serum was unavailable. In 2007, the grandfather (Y1) and mother (Y2W) had HBV DNA levels below the detection limit while the grandmother (Y1W) and granddaughter (Y3F) had genotype CD1. Father (Y2) and grandson (Y3M) had genotype D1.

### Phylogenetic Analysis of Family Y, Family Q and Family V

**Figure 2:**
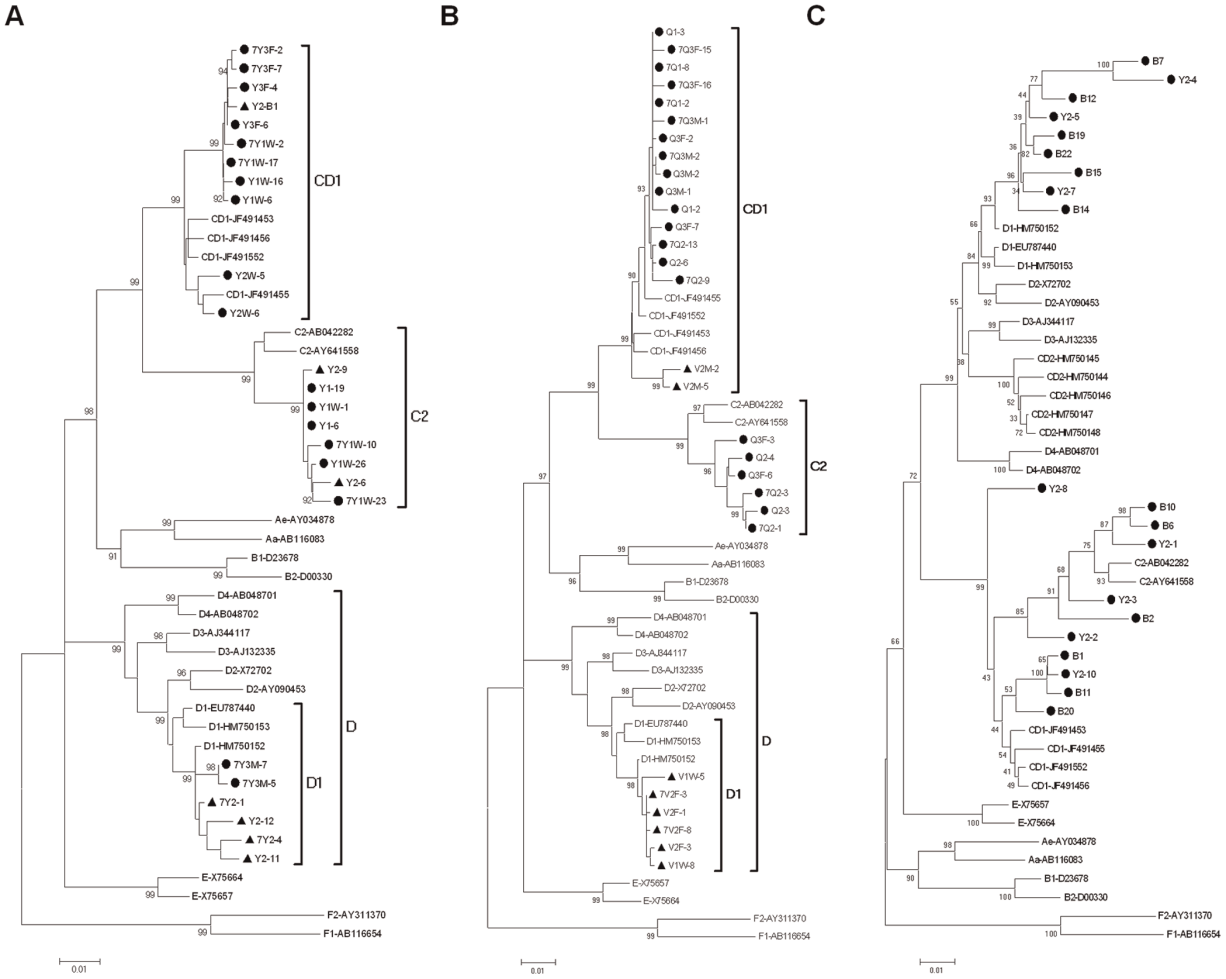
Phylogenetic tree construct by HBV nt 136-1110. (**A**) clones of family Y. Solid dots indicate the clones from Y1,Y1W,Y2W,Y3F and Y3M; Solid triangles indicate the clones from Y2. Family names starting with number 7 means the samples collected in 2007 otherwise in 2006. Novel recombinants of Y2 were excluded from the phylogenetic tree. (**B**) clones of family Q and family V. Solid dots indicate the clones from family Q; Solid triangles indicate the clones from family V. A family name starting with number 7 means the samples collected in 2007, otherwise, in 2006. (**C**) Novel recombinant clones of Y2. Solid dots indicate the clones from Y2.

A phylogenetic tree constructed from HBV nt 36-1110 from the clones of family Y is given (Figure 2A). The clones (dotted) of family Y exhibits three clusters on genotype C2, D1 and CD1.

The phylogenetic tree construct from HBV nt136-1110 from the clones of families Q and V is given (Figure 2B). The clones of family Q (indicated by black dots) exhibit two clusters of sub- genotypes C2, and CD1. The clones (indicated by black triangles) from family V exhibit two clusters of subgenotypes D1 and CD1.

A phylogenetic tree constructed from HBV nt 36-1110 of novel recombinants clones of Y2 is given in Figure 2C. The dotted clones are from Y2. The topology of phylogenetic tree with recombinants is totally different from typical trees. Recombinant sequences blurred the typical branch,in other words, blurred the typical genotype.

### Recombination and Crossover Analysis of Quasi-species of Y2

Results of recombination analysis of Y2 clones are as bellow: Three kinds of analytical methods certificated the same recombination map. The initial pictures of the three methods were all provided as supplemental figures. Recombination events detected by RDP software are shown in Figure S1, S2, and S3. Split phylogenetic trees constructed by MEGA software are shown in Figure S4, S5, and S6, (clone number and fragment used to construct tree are indicated beside each tree). Sequence alignments are shown in Figure S7, S8, and S9.

**Figure 3:**
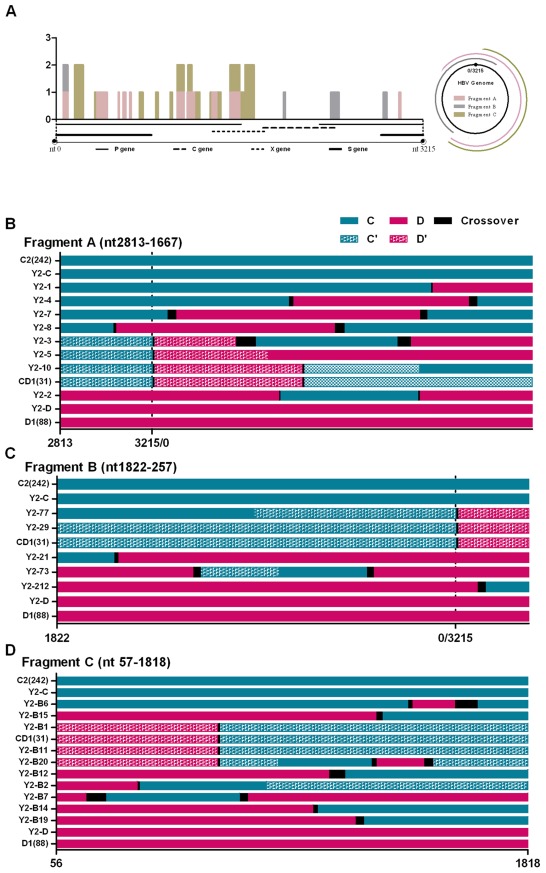
Alignment and recombination crossover regions found in Y2 clones. (**A**) Frequency and distribution of the recombination crossover regions found in Y2 clones along the HBV genome. The bars indicate the number of clones (y axis) showing recombination crossover regions at each site. The 1-3215 of x axis was consistent with the nt1-3215 of HBV genome. Different colors represent the sites find from clones of different PCR region: pink bars for fragment A, grey bars for fragment B and green bars for fragment C. (**B**) Alignment of fragment A (HBV nt 2813-0-1667). Y2-1′12: clones from fragment A of Y2 patients. (**C**) Alignment of fragment B (HBV nt 1822-0-257). Y2-21′212: clones from fragment B of Y2 patients. (**D**) Alignment of fragment C (HBV nt 57-1818) of Y2 clones. Y2-B1′B22: clones from fragment C of Y2 patients. The number on the x axis was consistent with the site of nucleotides of HBV genome. Solid green lines are genotype C2, solid pink lines are genotype D1, speckled green lines are the C2 component of genotype recombinant CD1 and speckled pink lines are the D1 component of recombinant genotype CD1. The black lines are sequence that is common to the recombining genotypes, and within which the recombination probably occurred. C2 (242) is the consensus sequence formed by 242 subgenotype C2 sequences from GenBank. D1 (88) is the consensus sequence formed by 88 subgenotype D1 sequences from GenBank. CD1 (33) is the consensus sequence formed by CD1 recombinant sequences from GenBank.

The region where recombination breakpoints had the highest probabilities was recognized as crossover region, which is a region that one parental genotype switches to another. Upstream sequence of crossover region will have specific mutation of one genotype but with no specific mutation of another, downstream just opposite. At the same time, these two genotypes should share same sequence at crossover region. We indicated the crossover region in direct alignment by black bars in Figure S3 initially and marked it in recombination map by colorful bars in Figure 3A and black bars in Figure 3B. The clonal sequences of 2006 showed 17 unique crossover regions in fragments A, B and C. We could not identify any common motif within these sequences that might suggest a common mechanism for crossovers in the HBV. The size of switch region share the same sequence are different in different strains, from 6–174 bp (6 bp for Y2M-2 clone in Figure S7 and 174 bp for Y2M-29 clone in Figure S8).

To illustrate the recombination map in a simple way. An abbreviated alignment of fragment A, B and C are shown in Figure 3B. Green and pink bars indicated the genotype C2 and D1 respectively. Black bars showed the crossover region. The aligned sequences provide a snapshot of the recombinant HBV strains. Genotype C2, D1 and CD1 recombinant clones of Y2 were all used as parental sequences to recombine with each other to form new recombinants. A series of novel recombinants were found in three fragments.

In 15 clones of fragment A, there were five genotype C (Y2-6,9,13,14,15,); two genotype D (Y2-11,12); one CD1 (Y2-10) and seven novel different C/D recombination (Y2-1,2,4,7,8,3,5).

In 16 clones of fragment B, there were four genotype C (Y2-23,71,78,75); seven genotype D (Y2-25, 27,79,76,72,22,210); one CD1 (Y2-29) and four novel C/D recombinants (Y2-212,21 73,77).

Of the 56 clones of fragment C(in which genotype B HBVDNA were added as an in-tube control to exclude the recombination by PCR procedure), there were 32 pure genotype B clones; nine genotype C clones(Y2-B10,B5,B8,B9,B13,B16.B17.B18,B24); five genotype D clones(Y2-B22,B3,B4,B21,B23), two CD1 clones (Y2-B1,B11) and eight novel C/D recombinants (Y2-B6,B7,B14,B15,B19,B2,B12,B20). No recombinants of genotype B were found.

## Discussion

Recombination is one of the major mechanisms contributing to the evolution of retroviruses [15]. Since the HBV has a reverse transcription step in its life cycle, it is conceivable that recombination also contributes to diversity in HBV genomes. Although just four cases were observed with mixed genotype infections, we obtained a snapshot of naturally occurring HBV recombinants generated in the absence of selection and after selection. Our result showed direct evidence of HBV recombination, with new information of recombining crossovers compared with similar studies [16], [17], [18], [19].

The recombination analysis of Y2 quasi-species showed variable types of recombinant between genotype C2, D1 and CD1 in 2006. Some studies show that hotspots of recombination most on the boundary of ORFs [12], [20]. Our results showed that two or more strains of HBV can recombine with each other at any region along the genome. Crossover regions can be hundreds or just several base pairs, The length of crossover region is depends on the location of it on HBV genome. If it is located in a conserved HBV region, for another word, where many different genotypes share the same sequence, the length of crossover region may be long. If it is located in a non-conserved region, it may be very short. At the same time, we found that the crossover region distributed totally at random on HBV genome. Consistent with our results, *in vitro* evidence showed the initial recombination events in a laboratory system of MHV were almost entirely randomly distributed along the sequence [21]. It was only after passage through cell culture, with the opportunity for selection to remove less fit variants, that crossover sites became “localized” to just a small area of the region examined. Crucially, they also suggested initial products of recombination may go undetected because of the action of strong purifying selection which will remove new, deleterious combinations of mutations. The conclusion is therefore an interpretation for the genotype change of Y2. The Y2 presented multiple strain infections of C2/D1/CD1 and many new recombinants with no obvious dominant genotype strain in 2006. After 18 months, however, all the type C2 and CD recombinant strains disappeared while the D strain became dominant. A similar case of mixed HBV genotype infection in which one genotype was lost and another prevailed was previously described in patients with HBeAg seroconversion [4], [22].

Epidemiologically, HBV genotype CD1 and C2 are the most common strains in ethnic minorities of northwest China with CD2 and D1 as minor strains. Precise mapping of recombination suggests C2 and D1 are parental sequences of CD1 and CD2 recombinants. Virological differences among HBV genotypes were demonstrated in vitroand in CHiM mice, with genotype C having a higher replication capacity than D [23]. Why does the replication-deficient genotype D virus predominate over replication-competent genotype C? As mixed HBV infections together with recombination are rare, we have little knowledge about i this situation. On the one hand, we know little about host impact on different genotypes and recombinants. On the other hand, we know little about interference and competition in the quasi-species of mixed infection. In vitro results showed the replication capacity of individual clone, exclude the influence of host and other strains of quasi-species. An example from a ChiM mice study showed that monoinfection of HBV/G in ChiM mice display a very slow replication while coinfection with HBV/A remarkably enhanced the replication of HBV/G. The replication of HBV/G is heavily dependent on coinfection with other genotypes. When HBV/G superinfected on other genotypes, a rapidly takes over of HBV/G from original genotype were observed, though they are indispensable [24]. This study confirms that in a mixed infection system of different genotypes, the replication capacity of a genotype may be different from that of monoinfection. At the same time, replication capacity is not the only factor to influence which strain will become dominant. Variable recombinants found in our study may be mechanistically capable of genetic exchange, but strong selection guaranteed the elimination of hybrid genomes. The mechanism of selection in mixed infection also needs more investigation.

We found mixed HBV genotypes infection with many novel recombinants at one point in time, but just one genotype was found 18 months later. This may indicate that the detectable mixed infection and recombination has a limited time window due to the sensitivity of detection or strong selection power of the host. That’s why in most studies, we can identify a major genotype in one patient. Even so, evolutionarily visible and invisible recombination of HBV could occur and play an important role by generating genetic variation or reducing mutational load. However, this study had limitation, because recombination signals were detected by RDP3 software and confirmed by split phylogenetic tree and alignment, indicating the recombinant or recombinant-like form should depend on the software. If we use another software, the results might be different.

Studies of HBV in endemic areas throughout the world have resulted in large numbers of full genome sequences available for phylogenetic analysis enabling the identification of novel, mosaic HBV genomes that appear to be the result of recombination between previously known sequences [7], [25], [26]. One of the most comprehensive analyses of putative HBV inter-genotype recombinants showed the existence of 24 phylogenetically independent HBV genomes involving all known human genotypes [27]. Some of these recombinants are unique to individual subjects, but some undergo expansion in specific populations and become recognized as new genotypes or subgenotypes [12], [28], [29], [30]. Four stages in the process of generating popular HBV recombinant genomes should be recognized. The first stage is the co-circulation of different HBV strains or genotypes in the same geographic area. The second is the existence of individuals who have been infected with more than one strain of HBV. The third is the generation of a novel recombinant strain(s) within an individual. The fourth is the selection of a recombined strain with the ability to replicate and be transmitted. Our data show the natural process of the formation and selection of recombination though the recombinant strains of Y2 that appeared in 2006 that were all removed from samples in 2007.

By using phylogenetic trees and homology calculations, HBV variants infecting humans are currently classified into ten genotypes that differ from each other in nucleotide sequence by 7.5 to 13% [2], [3]. There are some characteristic length differences between the genotypes that facilitates their detection and discrimination. However, as shown in Figure 2, existence of a recombinant makes the topology of the phylogenetic tree totally different from one with no recombinant. Recombinant strains obscured the definition of genotypes. Based on the algorithm creating a phylogenetic tree, sequences with high homologues cluster together. With the same logic, recombinants always clustered with the backbone parental sequence, in other words, with which they have high similarity with the larger proportion of the recombination region. Therefore, recombinants always seem to be a subgenotype of their backbone parental sequence. Similar to Y2-8 clone in Figure 2C, for recombinants with similar proportion of both parental genotypes, the sequence shows a divergent trend different from both parental genotypes.

Based on phylogenetic topology changes of different regions of HBV, it was hypothesized that some of the genotypes that are conventionally regarded as “pure,” actually were recombinant. Genotype E strains show evidence of recombination with genotype D at 1950–2500. new reported genotype “I” actually belongs to genotype C. Furthermore, Subgenotype Ba possesses the recombination with genotype C at 1740 to 2485 [31], [32], [33]. Recombinants comprising regions with different histories have important implications for the way we think about HBV evolution. It means that there is no single phylogenetic tree that can describe the evolutionary relationships between genotypes.

In conclusion, mixed HBV genotypes infection with many novel recombinants at one point in time ended up with just one genotype 18 months later in this study. This may indicate that the detectable mixed infection and recombination have a limited time window due to the sensitivity of detection or strong selection power of the host. Also, as the recombinant or recombinant-like nature of HBV precludes the possibility of a “true” phylogenetic taxonomy, a new standard may be required for classifying HBV sequences.

## Supporting Information

Figure S1 **Recombination map of fragment A created by RDP software.**
(TIF)

Figure S2 **Recombination map of fragment B created by RDP software.**
(TIF)

Figure S3 **Recombination map of fragment C created by RDP software.**
(TIF)

Figure S4 **Split phylogenetic trees constructed by MEGA software.** clone number and fragment used to construct trees are indicated beside each tree.
(TIF)

Figure S5 **Split phylogenetic trees constructed by MEGA software.** clone number and fragment used to construct trees are indicated beside each tree.
(TIF)

Figure S6 **Split phylogenetic trees constructed by MEGA software.** clone number and fragment used to construct trees are indicated beside each tree.
(TIF)

Figure S7 **Alignment of fragment A(HBV nt 2813-0-1667)of Y2 clones.** Deep green lines are genotype C2, deep pink lines are genotype D1, light green lines are the C2 component of genotype recombinant CD1 and light pink lines are the D1 component of recombinant genotype CD1. The black lines are sequence that is common to the recombining genotypes, and within which the recombination probably occurred. C2 (242): consensus sequence formed by 242 subgenotype C2 sequences from GenBank. D1 (88): consensus sequence formed by 88 subgenotype D1 sequences from GenBank. CD1 (33): consensus sequence formed by CD1 recombinant sequences from GenBank. Y2-1′12: clones from fragment A of Y2 patients.
(DOC)

Figure S8 **Alignment of fragment B(HBV nt 1822-0-257) of Y2 clones.** Deep green lines are genotype C2, deep pink lines are genotype D1, light green lines are the C2 component of genotype recombinant CD1, light pink lines are the D1 component of recombinant genotype CD1. The black lines are sequence that is common to the recombining genotypes, and within which the recombination probably occurred. C2 (242): consensus sequence formed by 242 subgenotype C2 sequences from GenBank. D1 (88): consensus sequence formed by 88 subgenotype D1 sequences from GenBank. CD1 (33): consensus sequence formed by CD1 recombinant sequences from GenBank. Y2-21′212: clones from fragment B of Y2 patients.
(DOC)

Figure S9 **Alignment of fragment C(HBV nt 57-1818) of Y2 clones.** Deep green lines are genotype C2, deep pink lines are genotype D1, light green lines are the C2 component of genotype recombinant CD1, light pink lines are the D1 component of recombinant genotype CD1. The black lines are sequence that is common to the recombining genotypes, and within which the recombination probably occurred. C2 (242): consensus sequence formed by 242 subgenotype C2 sequences from GenBank. D1 (88): consensus sequence formed by 88 subgenotype D1 sequences from GenBank. CD1 (33): consensus sequence formed by CD1 recombinant sequences from GenBank. B1̀B22: clones from fragment C of Y2 patients.
(DOC)
