# Supplementary figures and images for: Novel Evidence of HBV Recombination in Family Cluster Infections in Western China
Source: PLoS One. 2012 Jun 4;7(6):e38241. doi: 10.1371/journal.pone.0038241 (PMC3366946; doi:10.1371/journal.pone.0038241)

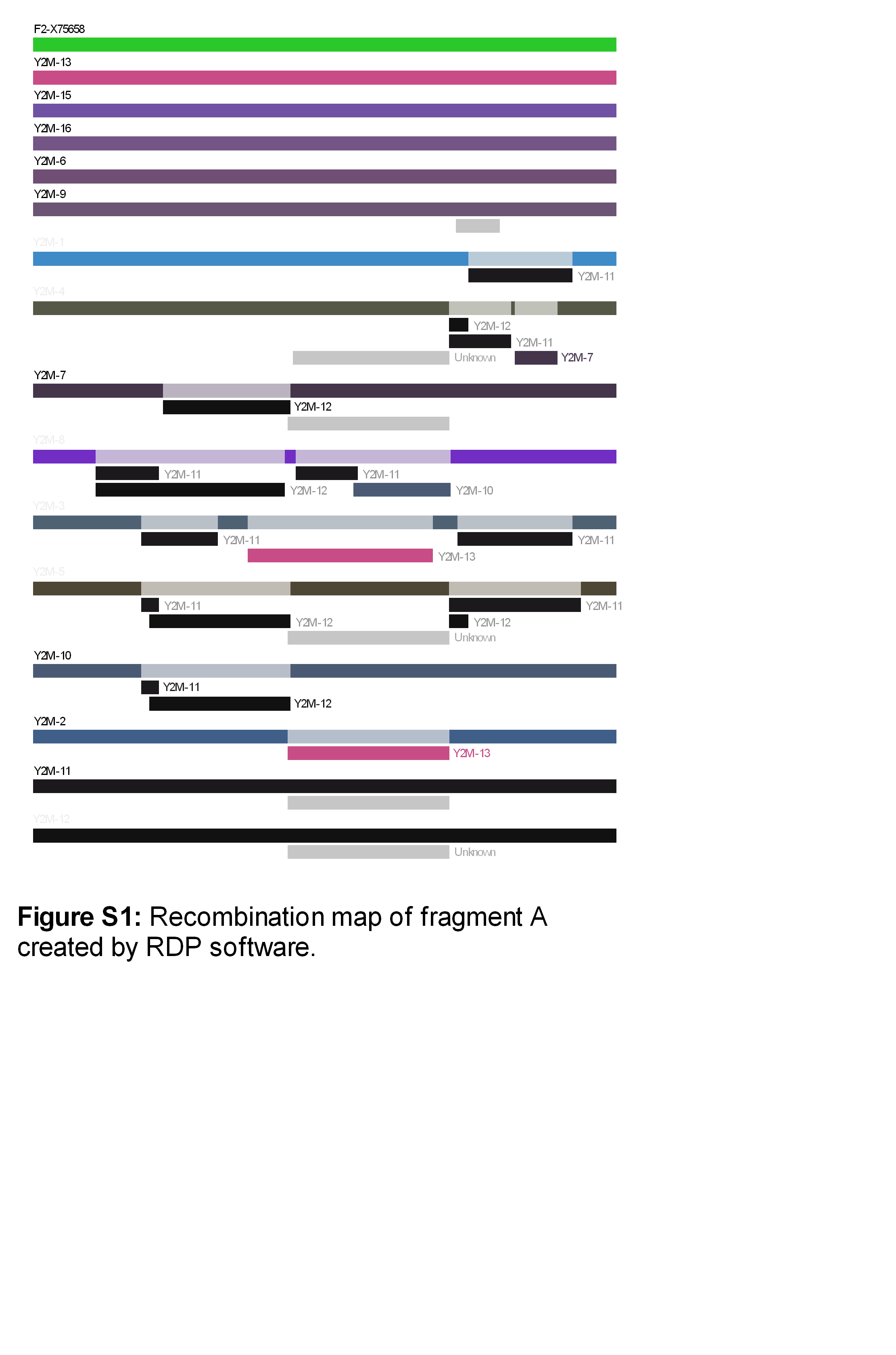

Supplement: Figure S1 — Recombination map of fragment A created by RDP software. (TIF) [file pone.0038241.s001.tif]

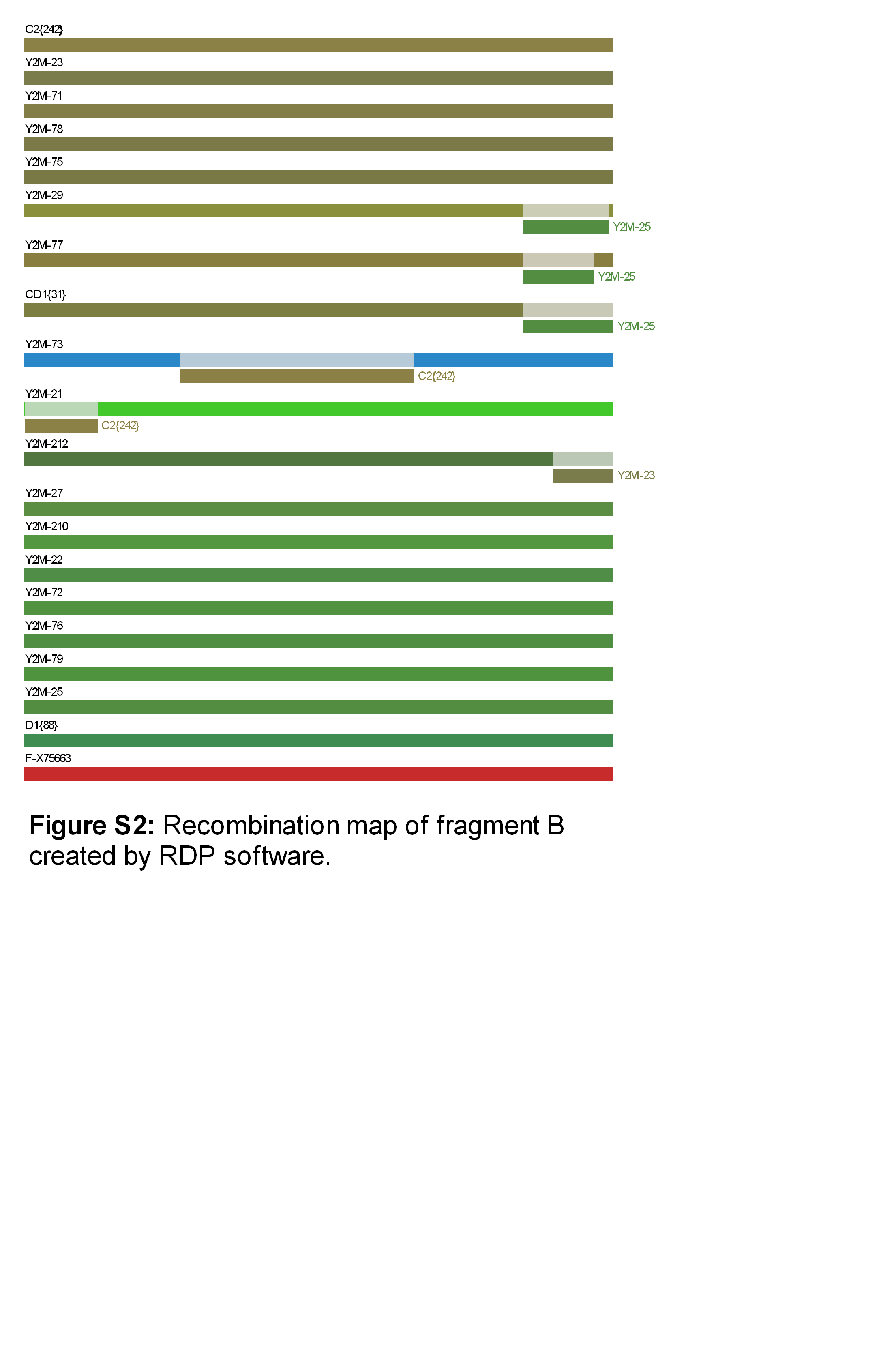

Supplement: Figure S2 — Recombination map of fragment B created by RDP software. (TIF) [file pone.0038241.s002.tif]

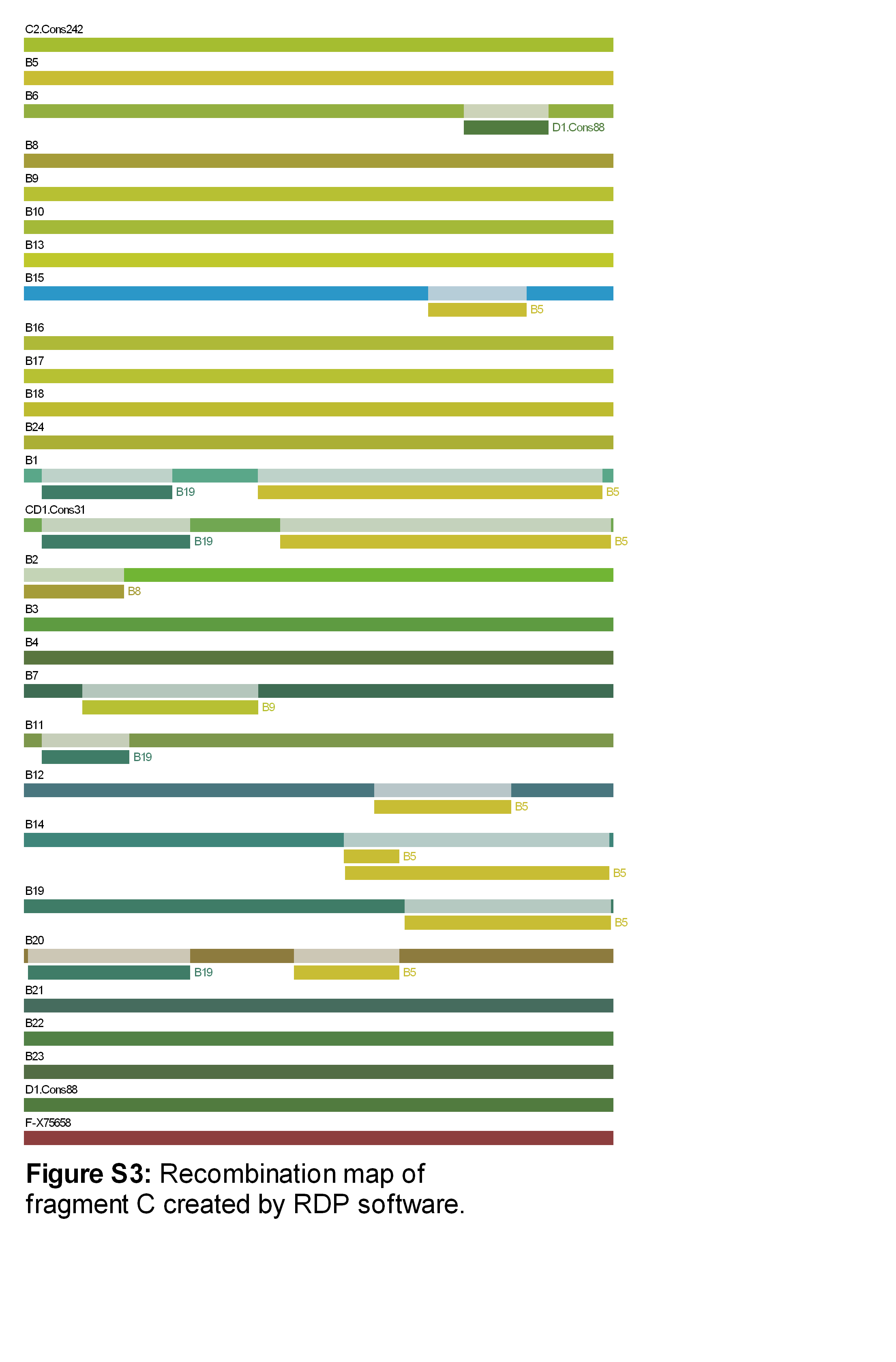

Supplement: Figure S3 — Recombination map of fragment C created by RDP software. (TIF) [file pone.0038241.s003.tif]

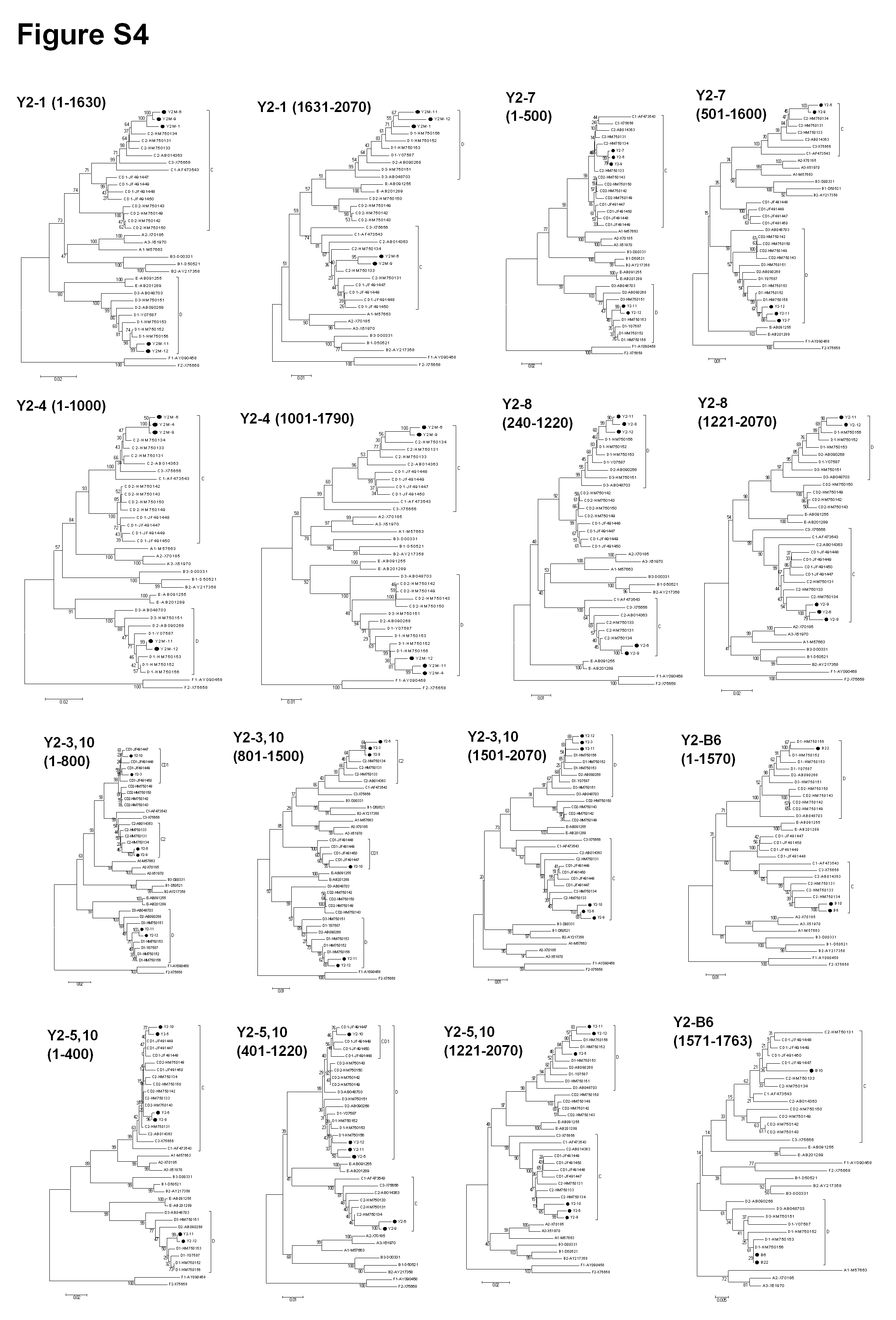

Supplement: Figure S4 — Split phylogenetic trees constructed by MEGA software. clone number and fragment used to construct trees are indicated beside each tree. (TIF) [file pone.0038241.s004.tif]

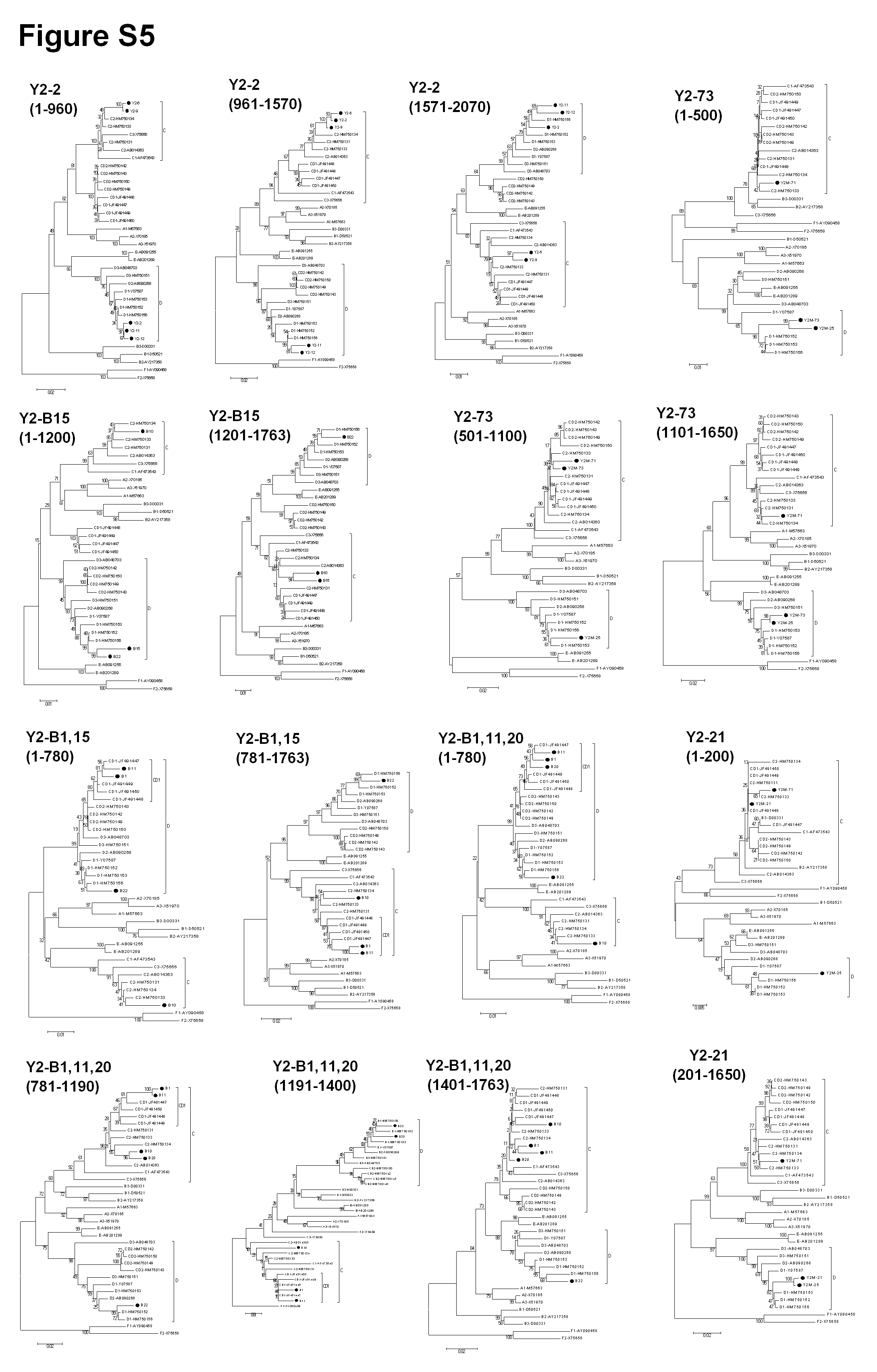

Supplement: Figure S5 — Split phylogenetic trees constructed by MEGA software. clone number and fragment used to construct trees are indicated beside each tree. (TIF) [file pone.0038241.s005.tif]

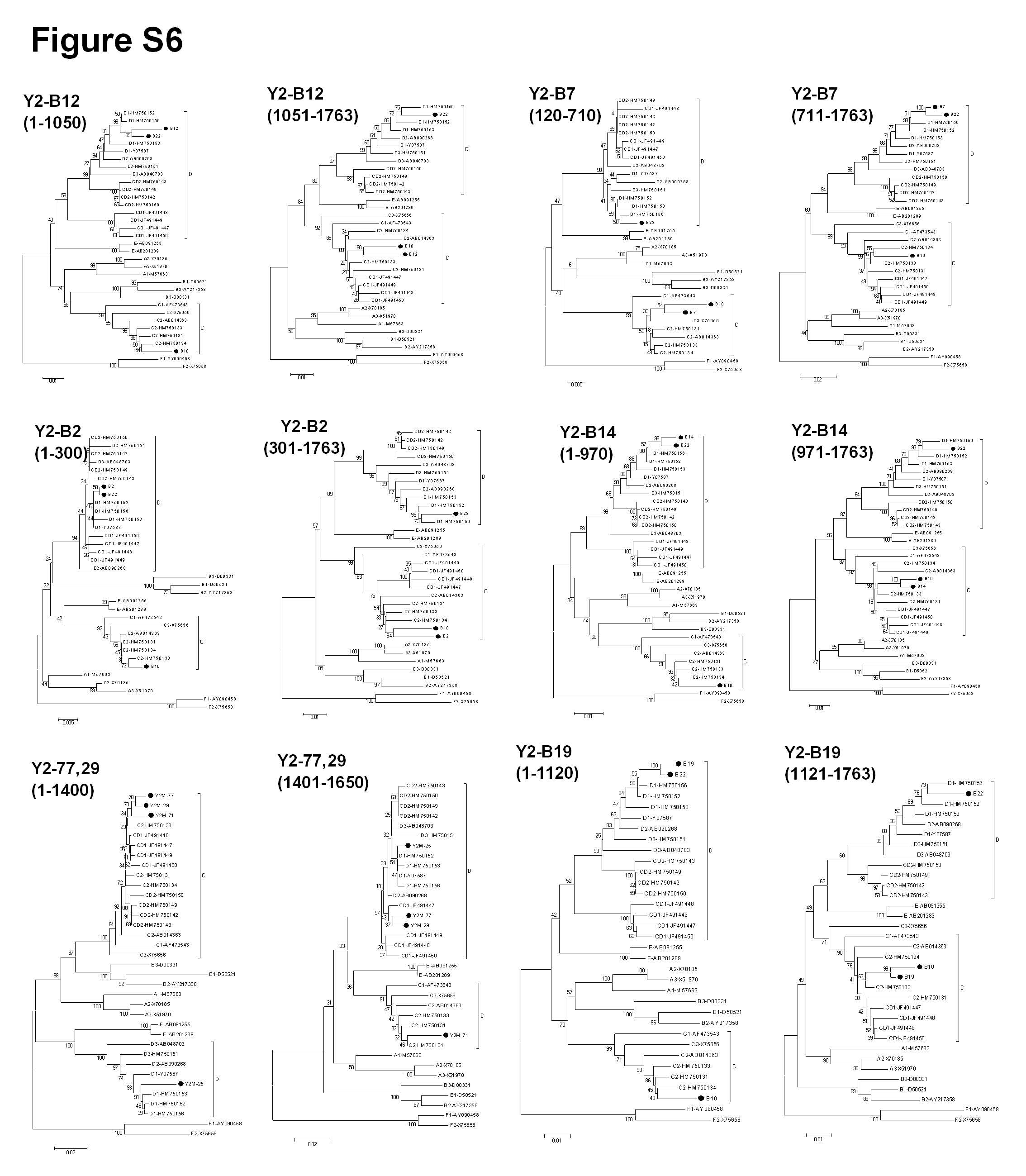

Supplement: Figure S6 — Split phylogenetic trees constructed by MEGA software. clone number and fragment used to construct trees are indicated beside each tree. (TIF) [file pone.0038241.s006.tif]
