# Supplementary material for: Novel Evidence of HBV Recombination in Family Cluster Infections in Western China
Source: PLoS One. 2012 Jun 4;7(6):e38241. doi: 10.1371/journal.pone.0038241 (PMC3366946; doi:10.1371/journal.pone.0038241)
Supplement: Figure S7 — Alignment of fragment A(HBV nt 2813-0-1667)of Y2 clones. Deep green lines are genotype C2, deep pink lines are genotype D1, light green lines are the C2 component of genotype recombinant CD1 and light pink lines are the D1 component of recombinant genotype CD1. The black lines are sequence that is common to the recombining genotypes, and within which the recombination probably occurred. C2 (242): consensus sequence formed by 242 subgenotype C2 sequences from GenBank. D1 (88): consensus sequence formed by 88 subgenotype D1 sequences from GenBank. CD1 (33): consensus sequence formed by CD1 recombinant sequences from GenBank. Y2-1′12: clones from fragment A of Y2 patients. (DOC) [file pone.0038241.s007.doc]

**~~~~~~~~~10~~~~~~~~20~~~~~~~~30~~~~~~~~40~~~~~~~~50~~~~~~~~60~~~~~~~~70~~~~~~~~80~~~~~~~~90~~~~~~~100~~~~~~~110~~~~~~~120~~~~~~~130~~~~~~~140~~~~~~~150~~~~~~~160~~~~~~~170~~~~~~~180~~~~~~~190~~~~~~~200~~~~~~~210~~~~~~~220~~~~~~~230~**

....|....|....|....|....|....|....|....|....|....|....|....|....|....|....|....|....|....|....|....|....|....|....|....|....|....|....|....|....|....|....|....|....|....|....|....|....|....|....|....|....|....|....|....|....|....|

**TGGGTCACCATATTCTTGGGAACAAGAGCTACAGCATGGGAGGTTGGTCTTCCAAACCTCGACAAGGCATGGGGACGAATCTTTCTGTTCCCAATCCTCTGGGATTCTTTCCCGATCACCAGTTGGACCCTGCGTTCGGAGCCAACTCAAACAATCCAGATTGGGACTTCAACCCCAACAAGGATCACTGGCCAGAGGCAAATCAGGTAGGAGCGGGAGCATTCGGGCCA C2(242)**

**.....................................................................C..................................................................T................G............................................................................ Y2M-6**

**...............................................................G.....C..................................................................T................G............................................................................ Y2M-9**

**.....................................................................C..................................................................T................G............................................................................ Y2M-1**

**.....................................................................C..................................................................T................G............................................................................ Y2M-4**

**.....................................................................C..................................................................T................G............................................................................ Y2M-7**

**.....................................................................C..................................................................T................G............................................................................ Y2M-8**

**.................................................................................................................................................................................G.................................................... Y2M-3**

**.........................................................................................................................................................................................................G....A....................... Y2M-5**

**......................................................................................................................................................................T............................................................... Y2M-10**

**...................................................................................................................................................................................................................................... CD1(31)**

**........................................--------------------------...-------.........CACCAG..G.....................C...........T..A..C...A....A...A..GCA....................T...........CAC.........C..C..CA..........T.............TG Y2M-2**

**........................................--------------------------...-------.........CACCAG........................C...........T..A..C...A....A...A..GCA....................T...........CAC.........C..C..CA..........T.............TG Y2M-11**

**.............................C..........--------------------------...-------.........CACCAG........................C...........T..A..C...A....A...A..GCA....................T...........CAC.........C..C..CA..........T.............TG Y2M-12**

**........................................--------------------------...-------.........CACCAG........................C...........T..A..C...A....A...A.CGCA....................T...........CAC.........C..C..CA..........T.............TG D1(88)**

**~~~~~~~~240~~~~~~~250~~~~~~~260~~~~~~~270~~~~~~~280~~~~~~~290~~~~~~~300~~~~~~~310~~~~~~~320~~~~~~~330~~~~~~~340~~~~~~~350~~~~~~~360~~~~~~~370~~~~~~~380~~~~~~~390~~~~~~~400~~~~~~~410~~~~~~~420~~~~~~~430~~~~~~~440~~~~~~~450~~~~~~~460**

....|....|....|....|....|....|....|....|....|....|....|....|....|....|....|....|....|....|....|....|....|....|....|....|....|....|....|....|....|....|....|....|....|....|....|....|....|....|....|....|....|....|....|....|....|....|

**GGGTTCACCCCACCACACGGCGGTCTTTTGGGGTGGAGCCCTCAGGCTCAGGGCATATTGACAACAGTGCCAGHAGCDCCTCCTCCTGCCTCCACCAATCGGCAGTCAGGAAGACAGCCTACTCCCATCTCTCCACCTCTAAGAGACAGTCATCCTCAGGCCATGCAGTGGAACTCCACAACATTCCACCAAGCTCTGCTAGAYCCCAGAGTGAGGGGCCTATAYTTTCC C2(242)**

**...........................................................A.......................................................T.........................................................T......................................A................. Y2M-6**

**...........................................................A.................................................................................................................T......................................A................. Y2M-9**

**...........................................................A.................................................................T...............................................T......................................A.....T........... Y2M-1**

**...........................................................A.................................................................................................................T......................................A................. Y2M-4**

**...........................................................A.................................................................................................................T.....G................................A................. Y2M-7**

**..............G.....A..C.................................C.ACA...CT........AA...G...........T........C.....C..G..G...........TC.G.........T.G...A...C........................T........C.........A...A..A...............A.....G.....C.. Y2M-8**

**........T.......................................................................................................................T.............................................C.......T.........A......A.........G.....A.....G.....C.. Y2M-3**

**........T............................................................................A................................................................................................C.........A......A.........G.....A.....G.....C.. Y2M-5**

**........T.........................................................................T..A....C...........................................................................................C.........A......A.........G.....A.....G.....C.. Y2M-10**

**........T.....V.......................................................................................................................................................................C.........A......A.........G.....A.....G.....C.. CD1(31)**

**..A...........G.....A..C.................................C.ACA...CT........AA...G...........T........C.....C..G..G...........TC.G.....G...T.G...A...C........................T........C.........A......A...............A.....G.....C.. Y2M-2**

**..A...........G.....A..C.................................C.ACA...CT........AA...G...........T........C.....C..G..G...........TC.G.........T.G...A...C........................T........C.........A......A...............A.....G.....C.. Y2M-11**

**..A...........G.....A..C.................................C.ACA...CT........AA...G...........T........C.....C..G..G...........TC.G.........T.G...A...C........................T........C.........A......AT..............A.....G.....C.. Y2M-12**

**..A...........G.....A..C.................................C.ACA...CT........AA...G...........T........C...........G........C..TC.G.........T.G...A...C.................................C.........A......A...............A.....G.....C.. D1(88)**

**~~~~~~~~470~~~~~~~480~~~~~~~490~~~~~~~500~~~~~~~510~~~~~~~520~~~~~~~530~~~~~~~540~~~~~~~550~~~~~~~560~~~~~~~570~~~~~~~580~~~~~~~590~~~~~~~600~~~~~~~610~~~~~~~620~~~~~~~630~~~~~~~640~~~~~~~650~~~~~~~660~~~~~~~670~~~~~~~680~~~~~~~690**

....|....|....|....|....|....|....|....|....|....|....|....|....|....|....|....|....|....|....|....|....|....|....|....|....|....|....|....|....|....|....|....|....|....|....|....|....|....|....|....|....|....|....|....|....|....|

**TGCTGGTGGCTCCAGTTCCGGAACAGTAAACCCTGTTCCGACTACTGCCTCACCCATATCGTCAATCTTCTCGAGGACTGGGGACCCTGCACCGAACATGGAGAVCACAACATCAGGATTCCTAGGACCCCTGCTCGTGTTACAGGCGGGGTTTTTCTTGTTGACAAGAATCCTCACAATACCACAGAGTCTAGACTCGTGGTGGACTTCTCTCAATTTTCTAGGGGGAG C2(242)**

**...............C..................................T.....C............................................................................................................................................................................. Y2M-6**

**...............C..................................T.....C.........................................................................................................................................G................................... Y2M-9**

**...............C..................................T.....C......................................................................................................................................G...................................... Y2M-1**

**...............C..................................T.....C............................................................................................................................................................................. Y2M-4**

**...............C...............................T...T.........................T............G.T..............TC..........................................................................G.............................................A Y2M-7**

**..................A............................T...T.........................T............G.T..............TC..........................................................................G.............................................A Y2M-8**

**..................A................................T.........................T............G................TC..........................................................................G.............................................A Y2M-3**

**..................A................................T...................T.....T............G................TC.....................................................................G....G.............................................A Y2M-5**

**..................A................................T...................T.....T............G................TC..........................................................................G.............................................A Y2M-10**

**..................A................................T.........................T............G................TC..........................................................................G.............................................A CD1(31)**

**..................A............................T...T.........................T............G.T..............TC..........................................................................G.............................................A Y2M-2**

**..................A............................T...T.........................T............G.T..............TC..........................................................................G.............................................A Y2M-11**

**..................A............................T...T.........................T............G.T..............TC..........................................................................G.............................................A Y2M-12**

**..................A............................T...T.A.......................T............G.T..............TC..........................................................................G.............................................A D1(88)**

**~~~~~~~~700~~~~~~~710~~~~~~~720~~~~~~~730~~~~~~~740~~~~~~~750~~~~~~~760~~~~~~~770~~~~~~~780~~~~~~~790~~~~~~~800~~~~~~~810~~~~~~~820~~~~~~~830~~~~~~~840~~~~~~~850~~~~~~~860~~~~~~~870~~~~~~~880~~~~~~~890~~~~~~~900~~~~~~~910~~~~~~~920**

....|....|....|....|....|....|....|....|....|....|....|....|....|....|....|....|....|....|....|....|....|....|....|....|....|....|....|....|....|....|....|....|....|....|....|....|....|....|....|....|....|....|....|....|....|....|

**CACCCACGTGTCCTGGCCAAAATTCGCAGTCCCCAACCTCCAATCACTCACCAACCTCTTGTCCTCCAATTTGTCCTGGCTATCGCTGGATGTGTCTGCGGCGTTTTATCATATTCCTCTTCATCCTGCTGCTATGCCTCATCTTCTTGTTGGTTCTTCTGGACTACCAAGGTATGTTGCCCGTTTGTCCTCTACTTCCAGGAACATCAACTACCAGCACGGGACCATGC C2(242)**

**.........................................................................................................................................................C....................................................A..........T............ Y2M-6**

**..............................................................................................................................................................................................................A..........T............ Y2M-9**

**..................................................................................................................................................................................A...........................A..........T............ Y2M-1**

**..............................................................................................................................C...............................................................................A..........T............ Y2M-4**

**.CA..GT.....T.............................................C.....C....C.........T....A...........................C.....................................................T...........................A........T.T.....C...........T..C... Y2M-7**

**.TA..GT.....T.............................................C..........C.........T................................C.....................................................T...........................A........T.T.....C...........T..C... Y2M-8**

**.TA..GT...C.T........................................................C.........T..............................................................................................................................A..........T............ Y2M-3**

**.TA..GT.....T.............................................C..........C.........T......................................................................................T...........................A........T.T.....C...........T..C... Y2M-5**

**.TA..GT.....T.............................................C..........C.........T......................................................................................T...........................A........T.T.....C........A.....C... Y2M-10**

**.TA..GT.....T.............................................C..........C.........T......................................................................................T...........................A........T.T.....C........A.....C... CD1(31)**

**.CA..GT.....T.............................................C..........C.........T................................C.....................................................T...........................A........T.G.....C...........T..C... Y2M-2**

**.CA..GT.....T.............................................C..........C.........T................................C.....................................................T...........................A........T.T.....C...........T..C... Y2M-11**

**.CA..GT.....T.............................................C..........C.........T....A...........................C.....................................................T...........................A........T.T.....C...........T..C... Y2M-12**

**.TA..GT.....T.............................................C..........C.........T................................C.....................................................T...........................A........T.T.....C.................. D1(88)**

**~~~~~~~~930~~~~~~~940~~~~~~~950~~~~~~~960~~~~~~~970~~~~~~~980~~~~~~~990~~~~~~~1000~~~~~~1010~~~~~~1020~~~~~~1030~~~~~~1040~~~~~~1050~~~~~~1060~~~~~~1070~~~~~~1080~~~~~~1090~~~~~~1100~~~~~~1110~~~~~~1120~~~~~~1130~~~~~~1140~~~~~~1150**

....|....|....|....|....|....|....|....|....|....|....|....|....|....|....|....|....|....|....|....|....|....|....|....|....|....|....|....|....|....|....|....|....|....|....|....|....|....|....|....|....|....|....|....|....|....|

**AAGACCTGCACGATTCCTGCTCAAGGAACCTCTATGTTTCCCTCTTGTTGCTGTACAAAACCTTCGGACGGAAACTGCACTTGTATTCCCATCCCATCATCCTGGGCTTTCGCAAGATTCCTATGGGAGTGGGCCTCAGTCCGTTTCTCCTGGCTCAGTTTACTAGTGCCATTTGTTCAGTGGTTCGTAGGGCTTTCCCCCACTGTTTGGCTTTCAGTTATATGGATGAT C2(242)**

**...........................C...........................T....................................................................................A........A................C......................................................G........ Y2M-6**

**...........................C..........................................................................................................................................C............................................................... Y2M-9**

**...........................C....................................................C.....................................................................................C.....................G......................................... Y2M-1**

**...........................C...............................................................C.........T..........G..A.......................C..........................................................................T............... Y2M-4**

**.GA..........C.......................A......C...........C.................T.....C........T...........T..........G..A.......................C.......................................................................................... Y2M-7**

**.GA..........C......................CA......C...........T........A........T.....C....................T..........G..A.......................C..........................................................................T............... Y2M-8**

**...........................C..........................................................................................................................................C.....................G......................................... Y2M-3**

**.GA..........C.......................A......C...........C.................T.....C....................T..........G..A.....................G.C..........................................................................T............... Y2M-5**

**.GA..........C.......................A......C..................A.......G..T.....C...............................G..A.......................C.........T................................................................................ Y2M-10**

**.GA..........C.......................A......C.............................T.....C...............................G..A.......................C.........T................................................................................ CD1(31)**

**.GA..........C.......................A............................................................................................A..................A................C......................................................G........ Y2M-2**

**.GA..........C.......................A......C...........C.................T.....C....................T..........G..A.......................C..........................................................................T............... Y2M-11**

**.GA..........C.......................A......C...........C.................T.....C....................T.............A.......................C.........................................................................................C Y2M-12**

**.GA..........C.......................A......C...........C.................T.....C....................Y..........G..A.......................C.......................................................................................... D1(88)**

**~~~~~~~~1160~~~~~~1170~~~~~~1180~~~~~~1190~~~~~~1200~~~~~~1210~~~~~~1220~~~~~~1230~~~~~~1240~~~~~~1250~~~~~~1260~~~~~~1270~~~~~~1280~~~~~~1290~~~~~~1300~~~~~~1310~~~~~~1320~~~~~~1330~~~~~~1340~~~~~~1350~~~~~~1360~~~~~~1370~~~~~~1380**

....|....|....|....|....|....|....|....|....|....|....|....|....|....|....|....|....|....|....|....|....|....|....|....|....|....|....|....|....|....|....|....|....|....|....|....|....|....|....|....|....|....|....|....|....|....|

**GTGGTATTGGGGGCCAAGTCTGTACAACATCTTGAGTCCCTTTTTACCTCTATTACCAATTTTCTTTTGTCTTTGGGTATACATTTGAACCCTAATAAAACCAAACGTTGGGGCTACTCCCTTAACTTCATGGGATATGTAATTGGAAGTTGGGGTACTTTACCNCAVGAACATATTGTACTAAAAMTCAAGCAATGTTTTCGNAAACTGCCTGTAAATAGACCTATTGA C2(242)**

**.................................................................................................................A............................................A............................................................C.......... Y2M-6**

**.................................................................................................................A............................................A............................................................C.......... Y2M-9**

**..............T..................................................................................................A.............................................C.G...................A.........................T...................... Y2M-1**

**.......................GTCG.....................G..G...........................................C.....GC.GA.A.....T..T..TT.AC.T........G.....C......T.C.AT..GT.A..G........T..C..CA...AG........AG........A.......T.....T..C..G........ Y2M-4**

**.................A.....GTCG.....................G..G...........................................C.....AC..A.A.....T.....TT.AC.T........G.....C.....G....AT..GT.A..G........T..C..CA...AG........AG........A.......T.....T..C..G........ Y2M-7**

**.......................GTCG.....................G..G.............................................................A............................................A............................................................C.......... Y2M-8**

**.................................................................................................................A............................................A............................................................C.......... Y2M-3**

**.......................GTCG.....................G..G...........................................C.....GC..A.A.....T..T..TT.AC.T........G.....C......T...AT..GT.A..G........T..C..CA...AG........AG........A.......T.....T..C..G........ Y2M-5**

**..................A.............................G..G...................C......................C.........G.............................C...A....................C.....................A.........................T...................... Y2M-10**

**..........................G.....................G..G......................................................................................A..........................................A.........................T...................... CD1(31)**

**.................................................................................................................A............................................A............................................................C.......... Y2M-2**

**.......................GTCG.....................G..G...........................................C.....GC..A.A.....T..T..TT.AC.T........G.....C......T...AT..GT.A..G........T..C..CA...AG........AG........A.......T.....T..C..G........ Y2M-11**

**..........................G.....................G..G..................................A........C.....AC..A.A.....T.....TT.AC.T........G.....C......T...AT..GT.A..G........T..C..CA...AG........AG........A.......T.....T..C..G..G..... Y2M-12**

**..........................G.....................G..G..................................A........C.....A...A.A.....T.....TT.AC.T........C.....C......T...AT..GT.A..G........T..C..CA...AG........AG........A.......T.....T..C..G........ D1(88)**

**~~~~~~~~1390~~~~~~1400~~~~~~1410~~~~~~1420~~~~~~1430~~~~~~1440~~~~~~1450~~~~~~1460~~~~~~1470~~~~~~1480~~~~~~1490~~~~~~1500~~~~~~1510~~~~~~1520~~~~~~1530~~~~~~1540~~~~~~1550~~~~~~1560~~~~~~1570~~~~~~1580~~~~~~1590~~~~~~1600~~~~~~1610**

....|....|....|....|....|....|....|....|....|....|....|....|....|....|....|....|....|....|....|....|....|....|....|....|....|....|....|....|....|....|....|....|....|....|....|....|....|....|....|....|....|....|....|....|....|....|

**TTGGAAAGTATGTCARAGAATTGTGGGTCTTTTGGGCTTTGCTGCCCCTTTTACACAATGTGGCTATCCTGCCTTVATGCCTTTATATGCATGTATACAATCTAAGCAGGCTTTCACTTTCTCGCCAACTTACAAGGCCTTTCTGTGTAAACAATATCTGMACCTTTACCCCGTTGCCCGGCAACGGTCAGGTCTCTGCCAAGTGTTTGCTGACGCAACCCCCACTGGAT C2(242)**

**....G...............................T................................A..............................................................................................................A..............T.................................. Y2M-6**

**....G.............G.................T...............................................................................................................................................A..............T.................................. Y2M-9**

**....................................T....................................................T..........................................................................................A..............T.................................. Y2M-1**

**C........T......C.T.................T.................G........T........T....A...C..G...........T...........................................................C..............................C.......G................................C. Y2M-4**

**C........T......C.T.................T.................G........T........T....A...C..G...........T...........................................................C..............................C.......G.................................. Y2M-7**

**....G.............G.................T...............................................................................................................................................A..............T.................................. Y2M-8**

**....G...............................T.......................................................................................................................C..............................C.......G................................C. Y2M-3**

**C........T......C.C.................T.................G........T........T........C..G...........T...........................................................C..............................C.......G................................C. Y2M-5**

**........................A................T..............................TC............................C..........................................................................A...........C.....T.................................. Y2M-10**

**...............................................................A........T.............................C............................................................................................................................... CD1(31)**

**....G...............................T...............................................................................................................................................A..............G................................C. Y2M-2**

**C........T......C.T.................T.................G........T........T....A...C..G...........T...........................................................C..............................C.......G....................G...........C. Y2M-11**

**C........T......C.T.................T.................G........T........T....A...C..G...........T...........................................................C..............................C.......G................................C. Y2M-12**

**.........C......C.T.................T..........................T........T........C..G...........T...........................................................C..............................C.......G................................C. D1(88)**

**~~~~~~~~1620~~~~~~1630~~~~~~1640~~~~~~1650~~~~~~1660~~~~~~1670~~~~~~1680~~~~~~1690~~~~~~1700~~~~~~1710~~~~~~1720~~~~~~1730~~~~~~1740~~~~~~1750~~~~~~1760~~~~~~1770~~~~~~1780~~~~~~1790~~~~~~1800~~~~~~1810~~~~~~1820~~~~~~1830~~~~~~1840**

....|....|....|....|....|....|....|....|....|....|....|....|....|....|....|....|....|....|....|....|....|....|....|....|....|....|....|....|....|....|....|....|....|....|....|....|....|....|....|....|....|....|....|....|....|....|

**GGGGCTTGGCNATHGGCCATCGGCGCATGCGTGGAACCTTTGTGGCTCCTCTGCCGATCCATACTGCGGAACTCCTAGCAGCTTGTTTTGCTCGCAGCCGGTCTGGAGCVAAACTTATCGGNACNGACAACTCTGTTGTCCTCTCTCGGAAATACACCTCCTTYCCATGGCTGCTMGGGTGTGCTGCCAACTGGATCCTGCGCGGGACGTCCTTTGTCTACGTCCCGTCG C2(242)**

**...................................................................A.....T..............C.........................................................................................A................................................... Y2M-6**

**...................................................................A.....T..............C..................................................................G......................A................................................... Y2M-9**

**.....................A...................CG....................................C..................A.............CA..C..........T...........T.....C..C.....T..A..G.................C......................................T............ Y2M-1**

**.........T...G.......A...................CG....................................C..................A.............CA..C..........T...........T.....C..C.....T..A..G.................C................................................... Y2M-4**

**...................................................................A.....T..............C.........................................................................................A...........................G....................... Y2M-7**

**...................................................................A.....T..............C.........................................................................................A................................................... Y2M-8**

**.........T...G.......A...................CG....................................C..................A.............CA..C..........T...........T.....C..C.....T..A..G.................C......................................T............ Y2M-3**

**.........T...G.......A...................CG.................................G..C..................A.............CA..C..........T...........T.....C..C.....T..A..G.................C......................................T............ Y2M-5**

**...................................................................A.....T..............C.........................................................................................A................................................... Y2M-10**

**...................................................................................................................C.................................................................................................................. CD1(31)**

**.........T...G....C..A...................CG....................................C..................A.............CA..C..........T...........T.....C..C.....T..A..G.................C......................................T............ Y2M-2**

**.........T...G.......A..A................CG.................................G..C..................A.............CA..C..........T...........T.....C..C.....T..A..G.................C......................................T............ Y2M-11**

**.........T...G.......A...................CG....................................C..............T...A.............CA..C..........T...........T.....C..C.....T..G..G.................C......................................T............ Y2M-12**

**.........T...G.......A...................CN....................................C..................A.............CA..C..........T...........T.....C..C.....T..A..G.................C......................................T............ D1(88)**

**~~~~~~~~1850~~~~~~1860~~~~~~1870~~~~~~1880~~~~~~1890~~~~~~1900~~~~~~1910~~~~~~1920~~~~~~1930~~~~~~1940~~~~~~1950~~~~~~1960~~~~~~1970~~~~~~1980~~~~~~1990~~~~~~2000~~~~~~2010~~~~~~2020~~~~~~2030~~~~~~2040~~~~~~2050~~~~~~2060~~~~~~2070**

....|....|....|....|....|....|....|....|....|....|....|....|....|....|....|....|....|....|....|....|....|....|....|....|....|....|....|....|....|....|....|....|....|....|....|....|....|....|....|....|....|....|....|....|....|....|

**GCGCTGAATCCCGCGGACGACCCGTCTCGGGGCCGTTTGGGNCTCTACCGTCCCCTTCTTCNTCTGCCGTTCCGGCCGACCACGGGGCGCACCTCTCTTTACGCGGTCTCCCCGTCTGTGCCTTCTCATCTGCCGGACCGTGTGCACTTCGCTTCACCTCTGCACGTCGCATGGAGACCACCGTGAACGCCCACCAGGTCTTGCCCAAGGTCTTACATAAGAGGACTCTT C2(242)**

**................................................................A..............................................................................................................................A...................................... Y2M-6**

**................................................................A...................................................................T..........................................................A...........................C.......... Y2M-9**

**.......................T...........C..........CT...........C...........T..A...............................A........................................................G................................AT...............................C Y2M-1**

**................................................................A..............................................................................................................................A...................................... Y2M-4**

**................................................................A........................................................................................C.G.......------------------------------------------------------------------- Y2M-7**

**................................................................A.....C.............................................................................................................................AT................................ Y2M-8**

**.......................T...........C..........CT...........C...........T..A...............................A..................C..........................................A...........................AT.........G.......T.............. Y2M-3**

**.......................T...........C..........CT...........C...........T..A...G...........................A.........................................................................................ATG............................... Y2M-5**

**......G.........................................................A..............................................................................................................................A...................................... Y2M-10**

**.............................................................................V........................................................................................................................................................ CD1(31)**

**.......................T...........C..........CT...........C...........T..................................A...................................................C.........A...........................AT................................ Y2M-2**

**.......................T...........C..........CT...........C...........T..A...............................A.........................................................................................ATG............................... Y2M-11**

**.......................T...........C..........CT...........C...........T..A...............................A.............................................................A...........................AT.................T.............. Y2M-12**

**.......................T...........C..........CT...........C...........T..A...............................A.........................................................................................AT................................ D1(88)**
