# Supplementary material for: Novel Evidence of HBV Recombination in Family Cluster Infections in Western China
Source: PLoS One. 2012 Jun 4;7(6):e38241. doi: 10.1371/journal.pone.0038241 (PMC3366946; doi:10.1371/journal.pone.0038241)
Supplement: Figure S8 — Alignment of fragment B(HBV nt 1822-0-257) of Y2 clones. Deep green lines are genotype C2, deep pink lines are genotype D1, light green lines are the C2 component of genotype recombinant CD1, light pink lines are the D1 component of recombinant genotype CD1. The black lines are sequence that is common to the recombining genotypes, and within which the recombination probably occurred. C2 (242): consensus sequence formed by 242 subgenotype C2 sequences from GenBank. D1 (88): consensus sequence formed by 88 subgenotype D1 sequences from GenBank. CD1 (33): consensus sequence formed by CD1 recombinant sequences from GenBank. Y2-21′212: clones from fragment B of Y2 patients. (DOC) [file pone.0038241.s008.doc]

**~~~~~~~~~10~~~~~~~~20~~~~~~~~30~~~~~~~~40~~~~~~~~50~~~~~~~~60~~~~~~~~70~~~~~~~~80~~~~~~~~90~~~~~~~100~~~~~~~110~~~~~~~120~~~~~~~130~~~~~~~140~~~~~~~150~~~~~~~160~~~~~~~170~~~~~~~180~~~~~~~190~~~~~~~200~~~~~~~**

**....|....|....|....|....|....|....|....|....|....|....|....|....|....|....|....|....|....|....|....|....|....|....|....|....|....|....|....|....|....|....|....|....|....|....|....|....|....|....|....|**

**TTTTCACCTCTGCCTAATCATCTCATGTTCATGTCCTACTGTTCAAGCCTCCAAGCTGTGCCTTGGGTGGCTTTGGGGCATGGACATTGACCCGTATAAAGAATTTGGAGCTTCTGTGGAGTTACTCTCTTTTTTGCCTTCTGACTTCTTTCCTTCTATTCGAGATCTCCTCGACACCGCCTCTGCTCTGTATCGGGAGG** **C2(242)**

**.................................................................................................................................G...........................................................A..........** **Y2M-71**

**..........................................................C......................................................................G...........................................................A..........** **Y2M-23**

**.................................................................................................................................G...........................................................A..........** **Y2M-75**

**..............................................................................................................................T..G...........................................................A..........** **Y2M-77**

**.................................................................................................................................G...........................................................A..........** **Y2M-29**

**........................................................................................................................................................................................................** **CD1(31)**

**........................................................................................................................................................................................................ Y2M-21**

**........................T.................................................A...............T..T........................C..........G.........G................AG.A........T..A..T........A.....A........A.** **Y2M-73**

**........................T.................................................A................G................................................................AG.A........T..A..T..T.....A...T.A........A.** **Y2M-27**

**........................T.................................................A...............T..T......................C.C..........G.....A...G................AG.A........T..A..T..T.....A...T.A........A.** **Y2M-212**

**........................T.................................................A...............T..T......................C.C..........G.........G................AG.A........T..A..T..T.....A...T.A........A.** **Y2M-25**

**........................T.................................................R...............T..T..................A................G..........................AG.A........T..A..T........A.....R........A.** **D1(88)**

**~~~~~~~~210~~~~~~~220~~~~~~~230~~~~~~~240~~~~~~~250~~~~~~~260~~~~~~~270~~~~~~~280~~~~~~~290~~~~~~~300~~~~~~~310~~~~~~~320~~~~~~~330~~~~~~~340~~~~~~~350~~~~~~~360~~~~~~~370~~~~~~~380~~~~~~~390~~~~~~~400~~~~~~~**

**....|....|....|....|....|....|....|....|....|....|....|....|....|....|....|....|....|....|....|....|....|....|....|....|....|....|....|....|....|....|....|....|....|....|....|....|....|....|....|....|**

**CCTTAGAGTCTCCGGAACATTGTTCACCTCACCATACAGCACTCAGGCAAGCTATTCTGTGTTGGGGTGAGTTGATGAATCTGGCCACCTGGGTGGGAAGTAATTTGGAAGACCCAGCATCCAGGGAATTAGTAGTCAGCTATGTCAATGTTAATATGGGCCTAAAAATCAGACAACTAYTGTGGTTTCACATTTCCTGT C2(242)**

**..........................................................C.............................T............................................................................................................... Y2M-71**

**..........................................................C.............................T............................................................................................................... Y2M-23**

**..........................................................C.............................T............................................................................................................... Y2M-75**

**..........................................................C.............................T....................G.......................................................................................... Y2M-77**

**..........................................................C.............................T............................................................................................................... Y2M-29**

**................................................................................T....................................................................................................................... CD1(31)**

**.............T..G...GT....T..........T..T...........A.....T........G..TC.A...TCG..A..............T...G.......C..T...A..........CC..........T.....T..CAC...............GT.T..G.......................T... Y2M-21**

**.............T..G...GT...............T..T...........A.....T........G..TC.A...TC...A..............TG..........C..T...A.......A..CC..........T.....T..CAC...............GT.T..G.......................T... Y2M-73**

**.............T..G...GT....T..........T..T...........A.....T........G..TC.A...TC...A..............T...........C..T...A..........CC..........T.....T..CAC...............GT.T..G.......................T... Y2M-27**

**.............T..G...GT....T..........T..T...........A.....T........G..AC.A....C...A..............TG..........C..T...A..........CCC.........T.....T..CAC...............GT.T..........................T... Y2M-212**

**.............T..G...GT....T..........T..T...........A.....T........G..TC.A...TC...A..............T...........C.TT...A..........CC..........T.....T..CAC...............GT.T..G.......................T... Y2M-25**

**.............T..G....................T..............A.....T..C.....G..AC.A....C...A..............TG.............T...AY.........CC..........T.....T..CAC...............GT....G.......................T... D1(88)**

**~~~~~~~~410~~~~~~~420~~~~~~~430~~~~~~~440~~~~~~~450~~~~~~~460~~~~~~~470~~~~~~~480~~~~~~~490~~~~~~~500~~~~~~~510~~~~~~~520~~~~~~~530~~~~~~~540~~~~~~~550~~~~~~~560~~~~~~~570~~~~~~~580~~~~~~~590~~~~~~~600~~~~~~~**

**....|....|....|....|....|....|....|....|....|....|....|....|....|....|....|....|....|....|....|....|....|....|....|....|....|....|....|....|....|....|....|....|....|....|....|....|....|....|....|....|**

**CTTACTTTTGGAAGAGAAACTGTTCTTGAGTATTTGGTGTCTTTTGGAGTGTGGATTCGCACTCCTCCNGCTTACAGACCACCAAATGCCCCTATCTTATCAACACTTCCGGAAACTACTGTTGTTAGACGACGAGGCAGGTCCCCTAGAAGAAGAACTCCCTCGCCTCGCAGACGAAGGTCTCAATCGCCGCGTCGCAG C2(242)**

**..............................................................A....................................................................................................................A.................... Y2M-71**

**..............................................................A....................................................................................................................A.................... Y2M-23**

**..............................................................A....................................................................................................................A.................... Y2M-75**

**..............................................................A...................T................................................................................................A.................... Y2M-77**

**..........................................................................T............................................................................................................................. Y2M-29**

**........................................................................................................................................................................................................ CD1(31)**

**..C..............C..G..CA.A.....C...........C.............................T..........................G...........G.........A......A..................................................................... Y2M-21**

**..C..............C..G..CA.A...............................................T................................................................................................C............................ Y2M-73**

**..C..............C..G..CA.A.................C.............................T..........................G...........G.........A......A..................................................................... Y2M-27**

**..C.............CC..G..CA.A.................C.............................T..........................GG..........G.........A......A..................................................................... Y2M-212**

**..C..............C..G..CA.A.................C.............................T..........................G...........G.........A......A..................................................................... Y2M-25**

**..C.................G..CA.A.................C.............................T......................................G...................................................................................... D1(88)**

**~~~~~~~~610~~~~~~~620~~~~~~~630~~~~~~~640~~~~~~~650~~~~~~~660~~~~~~~670~~~~~~~680~~~~~~~690~~~~~~~700~~~~~~~710~~~~~~~720~~~~~~~730~~~~~~~740~~~~~~~750~~~~~~~760~~~~~~~770~~~~~~~780~~~~~~~790~~~~~~~800~~~~~~~**

**....|....|....|....|....|....|....|....|....|....|....|....|....|....|....|....|....|....|....|....|....|....|....|....|....|....|....|....|....|....|....|....|....|....|....|....|....|....|....|....|**

**AAGATCTCAATCTCGGGAATCTCAATGTTAGTATCCCTTGGACTCATAAGGTGGGAAACTTTACTGGGCTTTATTCTTCTACTGTACCTGTCTTTAATCCTGAGTGGCAAACTCCCTCCTTTCCTMACATTCATTTACAGGAGGACATTATTAATAGATGTCAACAATATGTGGGCCCTCTTACAGTTAATGAAAAAAGG C2(242)**

**................A.......................................................................CA...................G.T........................................................T............................... Y2M-71**

**................A.......................................................................C....................G.T.....................C..................................T............................... Y2M-23**

**................A.......................................................................C....................G.T........................................................T............................... Y2M-75**

**................A....C......................................................................................................................................................A.....C..................... Y2M-77**

**...................................................C................................................................................G.......................................A.....C..................... Y2M-29**

**............................................................................................................................................................................A........................... CD1(31)**

**........C........G............A...T.............................G..............C.....T...........C...C.T...A....A.....T........T..A........CC.A........C..A.A....G......T...A.....A..C.....C.....G.....A Y2M-21**

**............................................................................................................................................................................A.....C..................... Y2M-73**

**.G......C........G............A...T........................C....G....................T.......C...C...C.T...A....A.....T........T..A........CC.A........C..A.A....G......T...A.....A..C.....C.....G.....A Y2M-27**

**.................G...........GA...T.............................G....................T...........C...C.T...A....A.....T........T..A........CC.A........C..A.A.C..G......T...A.....A..C.....C.....G.....A Y2M-212**

**........C........G............A...T.............................G....................T...........C...C.T...A....A.....T........T..A........CC.A........C..A.A....G......T...A.....A..C.....C.....G.....A Y2M-25**

**..................................T.............................G....................T...........C...C.T...A....A.....T........T..A........CC.A........C..A.A....G......T...A.....A..C.....C.....G.....A D1(88)**

**~~~~~~~~810~~~~~~~820~~~~~~~830~~~~~~~840~~~~~~~850~~~~~~~860~~~~~~~870~~~~~~~880~~~~~~~890~~~~~~~900~~~~~~~910~~~~~~~920~~~~~~~930~~~~~~~940~~~~~~~950~~~~~~~960~~~~~~~970~~~~~~~980~~~~~~~990~~~~~~~1000~~~~~~**

**....|....|....|....|....|....|....|....|....|....|....|....|....|....|....|....|....|....|....|....|....|....|....|....|....|....|....|....|....|....|....|....|....|....|....|....|....|....|....|....|**

**AGATTAAAATTAATTATGCCTGCTAGGTTCTATCCTAACCTTACCAAATATTTGCCCTTGGAHAAAGGCATTAAACCNTATTATCCTGAACATGCAGTTAATCATTACTTCAAAACTAGGCATTATTTACATACTCTGTGGAAGGCTGGCATTCTATATAAGAGAGAAACTACACGCAGCGCCTCATTTTGTGGGTCACC C2(242)**

**.............................T.................................................................................................................A........................................................ Y2M-71**

**.............................T........................................................................................................C.C......A........................................................ Y2M-23**

**..........................A............................................................................................................................................................................. Y2M-75**

**...............................................................................................................C.......................T........A....................................................... Y2M-77**

**...............................................................................................................C.......................T................................................................ Y2M-29**

**..........................................................................................T....................C........................................................................................ CD1(31)**

**...C.GC....G.................T.....A..GG....A...........A........G..T.................A......CT................C....C..A...........C.....A........G..T..AT................A....AT.........G.......A..... Y2M-21**

**.............................T.................................................................................................................A........................................................ Y2M-73**

**...C.GC....G.................T.....A..GG....A...........A........G..T.................A......CT................C....C..A...........C.....A........G..T..AT................A....AT.................A..... Y2M-27**

**...C.GC....G................CT.....A.-GG....A...........A........G..T.................A......CT................C....C..A...........C.....A........G..T..AT................A....AT.................A..... Y2M-212**

**...C.GC....G.................T.....A..GG....A...........A........G..T.................A......CT................C....C..A...........C.....A........G..T..AT................AT...AT.................A..... Y2M-25**

**...C.GC....G.................T.....A..NG................A........G..T.................A......CT................C....C..A...........C.....A........G..T..AT................A....AT....................... D1(88)**

**~~~~~~~~1010~~~~~~1020~~~~~~1030~~~~~~1040~~~~~~1050~~~~~~1060~~~~~~1070~~~~~~1080~~~~~~1090~~~~~~1100~~~~~~1110~~~~~~1120~~~~~~1130~~~~~~1140~~~~~~1150~~~~~~1160~~~~~~1170~~~~~~1180~~~~~~1190~~~~~~1200~~~~~~**

**....|....|....|....|....|....|....|....|....|....|....|....|....|....|....|....|....|....|....|....|....|....|....|....|....|....|....|....|....|....|....|....|....|....|....|....|....|....|....|....|**

**ATATTCTTGGGAACAAGAGCTACAGCATGGGAGGTTGGTCTTCCAAACCTCGACAAGGCATGGGGACGAATCTTTCTGTTCCCAATCCTCTGGGATTCTTTCCCGATCACCAGTTGGACCCTGCGTTCGGAGCCAACTCAAACAATCCAGATTGGGACTTCAACCCCAACAAGGATCACTGGCCAGAGGCAAATCAGGTA C2(242)**

**............................................................C..................................................................T................G....................................................... Y2M-71**

**............................................................C...........................................................................................................T............................... Y2M-23**

**............................................................C..................................................................T................G....................T.................................. Y2M-75**

**........................................................................................................................................................................T............................... Y2M-77**

**..........................................................................................................C............................................................................................. Y2M-29**

**........................................................................................................................................................................................................ CD1(31)**

**...............................--------------------------...-------.........CACCAG........................C...........T..A..C...A....A...A..GCA....................T...........CAC.........C..C..CA..... Y2M-21**

**............................................................C.............................................C...........T..A..C...A....A...A..GCA............A.......T...........CAC.........C..C..CA..... Y2M-73**

**...............................--------------------------...-------.........CACCAG........................C...........T..A..C...A....A...A..GCA...........A...A....T...........CAC.........C..C..CA..... Y2M-27**

**...............................--------------------------...-------.........CACCAG........................C...........T..A..C...A....A...A..GCA....................T...........CAC.........C..C..CA..... Y2M-212**

**...............................--------------------------...-------.........CACCAG........................C...........T..A..C...A....A...A..GCA....................T...........CAC.........C..C..CA..... Y2M-25**

**...............................--------------------------...-------.........CACCAG........................C...........T..A..C...A....A...A.CGCA....................T...........CAC.........C..C..CA..... D1(88)**

**~~~~~~~~1210~~~~~~1220~~~~~~1230~~~~~~1240~~~~~~1250~~~~~~1260~~~~~~1270~~~~~~1280~~~~~~1290~~~~~~1300~~~~~~1310~~~~~~1320~~~~~~1330~~~~~~1340~~~~~~1350~~~~~~1360~~~~~~1370~~~~~~1380~~~~~~1390~~~~~~1400~~~~~~**

**....|....|....|....|....|....|....|....|....|....|....|....|....|....|....|....|....|....|....|....|....|....|....|....|....|....|....|....|....|....|....|....|....|....|....|....|....|....|....|....|**

**GGAGCGGGAGCATTCGGGCCAGGGTTCACCCCACCACACGGCGGTCTTTTGGGGTGGAGCCCTCAGGCTCAGGGCATATTGACAACAGTGCCAGHAGCDCCTCCTCCTGCCTCCACCAATCGGCAGTCAGGAAGACAGCCTACTCCCATCTCTCCACCTCTAAGAGACAGTCATCCTCAGGCCATGCAGTGGAACTCCAC C2(242)**

**................................................................................A.................................................................................................................T..... Y2M-71**

**.............................T............................................................................A.......................................................................................T..... Y2M-23**

**.....................................G..........................................A.................................................................................................................T..... Y2M-75**

**.............................T............................................................................A............................................................................................. Y2M-77**

**.............................T............................................................................A............................................................................................. Y2M-29**

**.............................T.....V.................................................................................................................................................................... CD1(31)**

**.....T.............TG..A...........G.....A..C.................................C.ACA...CT........AA...G...........T........C.....C..G..G...........TC.G.........T.G...A...C........................T..... Y2M-21**

**.....T.....G.......TG..A...G.......G.....A..C.................................C.ACA...CT.A......AA...G...........T........C.....C..G..G...........TC.G.........T.G...A...C........................T..... Y2M-73**

**A....T.............TG..A...........G.....A..C.................................C.ACA...CT........AA...G...........T........C.....C..G..G...........TC.G.........T.G...A...C........................T..... Y2M-27**

**.....T.............TG..A...........G.....A..C..............T..................C.ACA...CT....T...AAC..G...........A.T......C...........G...........TC.G.........T.....A...C........................T..... Y2M-212**

**.....T.............TG..A...........G.....A..C.................................C.ACA...CT........AA...G...........T........C.....C..G..G...........TC.G.........T.G...A...C........................T..... Y2M-25**

**.....T.............TG..A...........G.....A..C.................................C.ACA...CT........AA...G...........T........C...........G........C..TC.G.........T.G...A...C.............................. D1(88)**

**~~~~~~~~1410~~~~~~1420~~~~~~1430~~~~~~1440~~~~~~1450~~~~~~1460~~~~~~1470~~~~~~1480~~~~~~1490~~~~~~1500~~~~~~1510~~~~~~1520~~~~~~1530~~~~~~1540~~~~~~1550~~~~~~1560~~~~~~1570~~~~~~1580~~~~~~1590~~~~~~1600~~~~~~**

**....|....|....|....|....|....|....|....|....|....|....|....|....|....|....|....|....|....|....|....|....|....|....|....|....|....|....|....|....|....|....|....|....|....|....|....|....|....|....|....|**

**AACATTCCACCAAGCTCTGCTAGAYCCCAGAGTGAGGGGCCTATAYTTTCCTGCTGGTGGCTCCAGTTCCGGAACAGTAAACCCTGTTCCGACTACTGCCTCACCCATATCGTCAATCTTCTCGAGGACTGGGGACCCTGCACCGAACATGGAGAVCACAACATCAGGATTCCTAGGACCCCTGCTCGTGTTACAGGCGG C2(242)**

**.................................A................................C..................................T.....C............................................................................................ Y2M-71**

**.................................A................................C..................................T.....C............................................................................................ Y2M-23**

**.................................A................................C..................................T.....C............................................................................................ Y2M-75**

**...C.........A......A.........G.....A.....G.....C....................A............................T...T...................T.....T............G................TC........................................** **Y2M-77**

**...C.........A......A.........G.....A.....G.....C....................A................................T...................T.....T............G................TC........................................** **Y2M-29**

**...C.........A......A.........G.....A.....G.....C....................A................................T.........................T............G................TC........................................** **CD1(31)**

**...C.........A......A...............A.....G.....C....................A............................T...T.........................T............G.T..............TC........................................ Y2M-21**

**...C.........A......A...............A.....G.....C....................A............................T...T.........................T............G.T..............TC........................................ Y2M-73**

**...C.........A......A...............A.....G.....C....................A............................T...T.........................T............G.T..............TC....T................................... Y2M-27**

**...C.........A......A...............A.....G.....C....................A...............................T.....C............................................................................................ Y2M-212**

**...C.........A......A...............A.....G.....C....................A............................T...T.........................T............G.T..............TC........................................ Y2M-25**

**...C.........A......A...............A.....G.....C....................A............................T...T.A.......................T............G.T..............TC........................................ D1(88)**

**~~~~~~~~1610~~~~~~1620~~~~~~1630~~~~~~1640~~~~~~1650**

**....|....|....|....|....|....|....|....|....|....|**

**GGTTTTTCTTGTTGACAAGAATCCTCACAATACCACAGAGTCTAGACTCG C2(242)**

**.................................................. Y2M-71**

**.................................................. Y2M-23**

**.................................................. Y2M-75**

**.................................................. Y2M-77**

**..................................G.....G......... Y2M-29**

**..................................G............... CD1(31)**

**..................................G............... Y2M-21**

**.....................C............................ Y2M-73**

**.................................................. Y2M-27**

**.................................................. Y2M-212**

**..................................G............... Y2M-25**

**..................................G............... D1(88)**
