# Supplementary material for: Novel Evidence of HBV Recombination in Family Cluster Infections in Western China
Source: PLoS One. 2012 Jun 4;7(6):e38241. doi: 10.1371/journal.pone.0038241 (PMC3366946; doi:10.1371/journal.pone.0038241)
Supplement: Figure S9 — Alignment of fragment C(HBV nt 57-1818) of Y2 clones. Deep green lines are genotype C2, deep pink lines are genotype D1, light green lines are the C2 component of genotype recombinant CD1, light pink lines are the D1 component of recombinant genotype CD1. The black lines are sequence that is common to the recombining genotypes, and within which the recombination probably occurred. C2 (242): consensus sequence formed by 242 subgenotype C2 sequences from GenBank. D1 (88): consensus sequence formed by 88 subgenotype D1 sequences from GenBank. CD1 (33): consensus sequence formed by CD1 recombinant sequences from GenBank. B1̀B22: clones from fragment C of Y2 patients. (DOC) [file pone.0038241.s009.doc]

**~~~~~~~~~10~~~~~~~~20~~~~~~~~30~~~~~~~~40~~~~~~~~50~~~~~~~~60~~~~~~~~70~~~~~~~~80~~~~~~~~90~~~~~~~100~~~~~~~110~~~~~~~120~~~~~~~130~~~~~~~140~~~~~~~150~~~~~~~160~~~~~~~170~~~~~~~180~~~~~~~190~~~~~~~200~~~~~~~**

....|....|....|....|....|....|....|....|....|....|....|....|....|....|....|....|....|....|....|....|....|....|....|....|....|....|....|....|....|....|....|....|....|....|....|....|....|....|....|....|

**CCTGCTGGTGGCTCCAGTTCCGGAACAGTAAACCCTGTTCCGACTACTGCCTCACCCATATCGTCAATCTTCTCGAGGACTGGGGACCCTGCACCGAACATGGAGAVCACAACATCAGGATTCCTAGGACCCCTGCTCGTGTTACAGGCGGGGTTTTTCTTGTTGACAAGAATCCTCACAATACCACAGAGTCTAGACTC** **C2(242)**

**....................................................T.....C............................................................................................................................................. B10**

**....................................................T.....C..............................C.............................................................................................................. B6**

**....................................................T.....C..............................................................................................................................G.............. B15**

**.....................................................T.........................T............G...........A....TC..........................................................................G..............** **B1**

**....................A................................T.........................T............G................TC..........................................................................G..............** **CD1(31)**

**....................A................................T...................T.....T............G................TC..........................................................................G..............** **B11**

**T...................A............................T...T.........................T............G.T..............TC..........................................................................G.............. B20**

**....................A...............A................T...................T.....T............G................TC......................................................................................... B12**

**....................A............................T...T.........................T............G.T..............TC..........................................................................G.............. B2**

**....................A............................T...T.........................T............G.T..............TC......................................................................................... B7**

**....................A............................T...T.........................T............G.T..............TC..G.......................................................................G.............A B14**

**....................A............................T...T.........................T............G.T..............TC..........................................................................G.............. B19**

**....................A............................T...T.........................T............G.T..............TC..........................................................................G.............. B22**

**....................A............................T...T.A.......................T............G.T..............TC..........................................................................G.............. D1(88)**

**~~~~~~~~210~~~~~~~220~~~~~~~230~~~~~~~240~~~~~~~250~~~~~~~260~~~~~~~270~~~~~~~280~~~~~~~290~~~~~~~300~~~~~~~310~~~~~~~320~~~~~~~330~~~~~~~340~~~~~~~350~~~~~~~360~~~~~~~370~~~~~~~380~~~~~~~390~~~~~~~400~~~~~~~**

....|....|....|....|....|....|....|....|....|....|....|....|....|....|....|....|....|....|....|....|....|....|....|....|....|....|....|....|....|....|....|....|....|....|....|....|....|....|....|....|

**GTGGTGGACTTCTCTCAATTTTCTAGGGGGAGCACCCACGTGTCCTGGCCAAAATTCGCAGTCCCCAACCTCCAATCACTCACCAACCTCTTGTCCTCCAATTTGTCCTGGCTATCGCTGGATGTGTCTGCGGCGTTTTATCATATTCCTCTTCATCCTGCTGCTATGCCTCATCTTCTTGTTGGTTCTTCTGGACTACC C2(242)**

**........................................................................................................................................................................................................ B10**

**........................................................................................................................................................................................................ B6**

**...............................A.CA..GT.....T....................................G........C..........C.........T................................C........................................C............T. B15**

**...............................A.TA..GT.....T.............................................C..........C.........T......................................................................................T. B1**

**...............................A.TA..GT.....T.............................................C..........C.........T......................................................................................T. CD1(31)**

**...............................A.TA..GT.....T...................T.........................C..........C.........T......................................................................................T. B11**

**...............................A.TA..GT.....T.............................................C..........C.........T......................................................................................T. B20**

**............................................T.............................................C..........C.........T......................................................................................T. B12**

**...............................A.CA..GT.....T.............................................C............................................................................................................. B2**

**........................................................................................................................................................................................................ B7**

**............C..................A.TA..GT.....T.............................................C..........C.........T......................................................................................T. B14**

**...............................A.CA..GT.....T.............................................C..........C.........T....A...........................C.....................................................T. B19**

**...............................A.CA..GT.....T.............................................C..........C.........T................................C.....................................................T. B22**

**...............................A.TA..GT.....T.............................................C..........C.........T................................C.....................................................T. D1(88)**

**~~~~~~~~410~~~~~~~420~~~~~~~430~~~~~~~440~~~~~~~450~~~~~~~460~~~~~~~470~~~~~~~480~~~~~~~490~~~~~~~500~~~~~~~510~~~~~~~520~~~~~~~530~~~~~~~540~~~~~~~550~~~~~~~560~~~~~~~570~~~~~~~580~~~~~~~590~~~~~~~600~~~~~~~**

....|....|....|....|....|....|....|....|....|....|....|....|....|....|....|....|....|....|....|....|....|....|....|....|....|....|....|....|....|....|....|....|....|....|....|....|....|....|....|....|

**AAGGTATGTTGCCCGTTTGTCCTCTACTTCCAGGAACATCAACTACCAGCACGGGACCATGCAAGACCTGCACGATTCCTGCTCAAGGAACCTCTATGTTTCCCTCTTGTTGCTGTACAAAACCTTCGGACGGAAACTGCACTTGTATTCCCATCCCATCATCCTGGGCTTTCGCAAGATTCCTATGGGAGTGGGCCTCA C2(242)**

**......................................A..........T.......................................C................................T............................................................................. B10**

**......................................A..........T..................C....................C.............................................................................................................. B6**

**..........................A........T.T.....C...........T..C....GA..........C............G..........A......C...........C.................T.....C....................T..........G..A...................... B15**

**..........................A........T.T.....CT.......A.....C....GA..........C.......................A......C..................A..........T.....C...............................G..A...................... B1**

**..........................A........T.T.....C........A.....C....GA..........C.......................A......C.............................T.....C...............................G..A...................... CD1(31)**

**.................................A.T.T.....C........A.....C....GA..........C.......................A......C..................A..........T.....C...............................G..A...................... B11**

**..........................A........T.T.....C........A.....C....GA..........C.......................A......C...............T..A..........T...T.C...............................G..A...................... B20**

**..........................A........T.T.....C...........T..C....GA..........C.......................A......C...........C.................T.....C....................T..........G..A...................... B12**

**......................................A..........T.......................................C.............................................................................................................. B2**

**......................................A..........T.........................C.......................A......C............................................................................................. B7**

**..........................A........T.T.....C...........T..C....GA..........C.......................A......C...........C.................T.....C....................T..........G..A...................... B14**

**..........................A........T.T.....C...........T..C....GA..........C.......................A......C...........C.................T.....C....................T..........G..A.................T.... B19**

**..........................A........T.T.....C...........T..C....GA..........C.......................A......C...........T.................T.....C....................T..........G..A...................... B22**

**..........................A........T.T.....C...................GA..........C.......................A......C...........C.................T.....C....................Y..........G..A...................... D1(88)**

**~~~~~~~~610~~~~~~~620~~~~~~~630~~~~~~~640~~~~~~~650~~~~~~~660~~~~~~~670~~~~~~~680~~~~~~~690~~~~~~~700~~~~~~~710~~~~~~~720~~~~~~~730~~~~~~~740~~~~~~~750~~~~~~~760~~~~~~~770~~~~~~~780~~~~~~~790~~~~~~~800~~~~~~~**

....|....|....|....|....|....|....|....|....|....|....|....|....|....|....|....|....|....|....|....|....|....|....|....|....|....|....|....|....|....|....|....|....|....|....|....|....|....|....|....|

**GTCCGTTTCTCCTGGCTCAGTTTACTAGTGCCATTTGTTCAGTGGTTCGTAGGGCTTTCCCCCACTGTTTGGCTTTCAGTTATATGGATGATGTGGTATTGGGGGCCAAGTCTGTACAACATCTTGAGTCCCTTTTTACCTCTATTACCAATTTTCTTTTGTCTTTGGGTATACATTTGAACCCTAATAAAACCAAACGT C2(242)**

**............................C....C.....................................................................A................................................................................................ B10**

**.C.........A................C......................................................G....................-............................................................................................... B6**

**.C..........................................................................T................................A.....GTCG.....................G..G...........................................C.....AC..A.A B15**

**.C.........T................................................................................................................................G..G..........................................C.........G... B1**

**.C.........T..........................................................................................................G.....................G..G........................................................ CD1(31)**

**.C.........T.............................................................................................................C..................G..G..........................................C.........G... B11**

**.C.........T................................................................................................................................G..G..........................................C.........G... B20**

**.C.....C....................................................................T.............................T..A.....GTCG.....................G..G...........................................C.....AC..A.A B12**

**...........A................C......................................................G......................................................................................................C.........G... B2**

**...........A................C......................................................G...............................GTCG.....................G..G...........................................C.....GC..A.A B7**

**.C..........................................................................T................................A.....GTCG.....................G..G...........................................C.....AC..A.A B14**

**.C...............................................C.............T............T......................................GTCG.....................G..G...........................................C.....GC..A.A B19**

**.C..........................................................................T......................................GTCG.....................G..G....................C......................C.....GC..A.A B22**

**.C....................................................................................................................G.....................G..G..................................A........C.....A...A.A D1(88)**

**~~~~~~~~810~~~~~~~820~~~~~~~830~~~~~~~840~~~~~~~850~~~~~~~860~~~~~~~870~~~~~~~880~~~~~~~890~~~~~~~900~~~~~~~910~~~~~~~920~~~~~~~930~~~~~~~940~~~~~~~950~~~~~~~960~~~~~~~970~~~~~~~980~~~~~~~990~~~~~~~1000~~~~~~**

....|....|....|....|....|....|....|....|....|....|....|....|....|....|....|....|....|....|....|....|....|....|....|....|....|....|....|....|....|....|....|....|....|....|....|....|....|....|....|....|

**TGGGGCTACTCCCTTAACTTCATGGGATATGTAATTGGAAGTTGGGGTACTTTACCNCAVGAACATATTGTACTAAAAMTCAAGCAATGTTTTCGNAAACTGCCTGTAAATAGACCTATTGATTGGAAAGTATGTCARAGAATTGTGGGTCTTTTGGGCTTTGCTGCCCCTTTTACACAATGTGGCTATCCTGCCTTVAT C2(242)**

**.....A............................................A...................................................T........C..............G...............................T......................................... B10**

**.....A.G..........................................A............................................................C..............G...............................T......................................... B6**

**.....T.....TT.AC.T...G....G.....C..........AT..GT.A..G........T..C..CA...AG........AG........A.......T.....T..C..G........C........T......C.T.................T......A..........G........T........T..... B15**

**..........................C...A....................C...............C.....A.........................T..............................................A................T..............................TC.... B1**

**..............................A..........................................A.........................T.....................................................................................A........T..... CD1(31)**

**..........................C...A....................C.....................A.........................T..............................................A................T..............................TC.... B11**

**....-.....................C...A...................A............................................................C..............G...............................T......................................... B20**

**.....T.....TT.AC.T........G.....C......T...AT..GT.A..G........T..C..CA...AG.....T..AG........A.......T.....T..C..G........C........T......C.C.................T.................G........T........T..... B12**

**..........................C...A....................C.....................A.........................T..............................................A..........A.....T............G.................TC.... B2**

**.....T..T..TT.AC.T........G.....C......T...AT..GT.A..G........T..C..CA...AG........AG........A.......T.....T..C..G........C........T......C.T.................T.................G........T........T....A B7**

**.....T.....TT.AC.T........G.....C..........AT..GT.A..G........T..C..CA...AG........AG........A.......T.....T..C..G........C........T......C.T.................T......................................... B14**

**.....T..T..TT.AC.T........G.....C......T...AT..GC.A..G........T..C..CA...AG........AG........A.......T.....T..C..G........C...............C.T.................T.................G........T........T....A B19**

**.....T..T..TT.AC.T........G.....C......T...AT..GT.A..G........T..C..CA...AG........AG........A.......T.....T..C..G........C........T......C.T.................T.................G........T........T....A B22**

**.....T.....TT.AC.T........C.....C......T...AT..GT.A..G........T..C..CA...AG........AG........A.......T.....T..C..G.................C......C.T.................T..........................T........T..... D1(88)**

**~~~~~~~~1010~~~~~~1020~~~~~~1030~~~~~~1040~~~~~~1050~~~~~~1060~~~~~~1070~~~~~~1080~~~~~~1090~~~~~~1100~~~~~~1110~~~~~~1120~~~~~~1130~~~~~~1140~~~~~~1150~~~~~~1160~~~~~~1170~~~~~~1180~~~~~~1190~~~~~~1200~~~~~~**

....|....|....|....|....|....|....|....|....|....|....|....|....|....|....|....|....|....|....|....|....|....|....|....|....|....|....|....|....|....|....|....|....|....|....|....|....|....|....|....|

**GCCTTTATATGCATGTATACAATCTAAGCAGGCTTTCACTTTCTCGCCAACTTACAAGGCCTTTCTGTGTAAACAATATCTGMACCTTTACCCCGTTGCCCGGCAACGGTCAGGTCTCTGCCAAGTGTTTGCTGACGCAACCCCCACTGGATGGGGCTTGGCNATHGGCCATCGGCGCATGCGTGGAACCTTTGTGGCTC C2(242)**

**......................................................................................................A..............T.................................................................................. B10**

**......................................................................................................A..............T.................................................................................. B6**

**...CC.G.T...G.....T...........................................................C..............................C.......G................................C..........T...G.......A...................CG..... B15**

**........................C......................................................................................C....................C.............................................C.A................... B1**

**........................C............................................................................................................................................................................... CD1(31)**

**........................C..........-...........................................................................C..................................................................C.A...........A....... B11**

**..........................................................-...........................................A..............T....................G......................................................CG..... B20**

**...C..G...........T...................................................................................A..............T.................................................................................. B12**

**........................C...........................CG.........................................................C..................................................................C.A................... B2**

**...C..G...........T...........................................................C..............................C.......G................................C..........T...G.......A...................CG..... B7**

**......................................................................................................A...T..........T.................................................................................. B14**

**...C..G...........T...........................................................C..............................C.......G.................................................................................. B19**

**...C..G...........T...........................................................C..............................C.......G.............T......G...........C..........T...G.......A...................CG..... B22**

**...C..G...........T...........................................................C..............................C.......G................................C..........T...G.......A...................CN..... D1(88)**

**~~~~~~~~1210~~~~~~1220~~~~~~1230~~~~~~1240~~~~~~1250~~~~~~1260~~~~~~1270~~~~~~1280~~~~~~1290~~~~~~1300~~~~~~1310~~~~~~1320~~~~~~1330~~~~~~1340~~~~~~1350~~~~~~1360~~~~~~1370~~~~~~1380~~~~~~1390~~~~~~1400~~~~~~**

....|....|....|....|....|....|....|....|....|....|....|....|....|....|....|....|....|....|....|....|....|....|....|....|....|....|....|....|....|....|....|....|....|....|....|....|....|....|....|....|

**CTCTGCCGATCCATACTGCGGAACTCCTAGCAGCTTGTTTTGCTCGCAGCCGGTCTGGAGCVAAACTTATCGGNACNGACAACTCTGTTGTCCTCTCTCGGAAATACACCTCCTTYCCATGGCTGCTMGGGTGTGCTGCCAACTGGATCCTGCGCGGGACGTCCTTTGTCTACGTCCCGTCGGCGCTGAATCCCGCGGAC C2(242)**

**...................A.....T..............C.................G...............................................................T.......A..................................................................... B10**

**...................A.....T..............C.........................................................................................C......................................T.............................. B6**

**...................A.....T..............C.........................................................................................A..................................................................... B15**

**.................T.................................................C.............................................................................................................................T...... B1**

**...................................................................C.................................................................................................................................... CD1(31)**

**...................................................................C.................................................................................................................A.................. B11**

**...............................C..C...............A.............CA..C..........T..........CT.....C..C.....T..A..G.................C......................................T.............................. B20**

**...................A.....T..............C.....................................................T...................................A..................................................................... B12**

**.................T..........................................T......C.................................................................................................................................... B2**

**...............................C..................A.............CA..C..........T...........T.....C..C.....T..A..G.................A......................................T.............................. B7**

**...C...............A.....T..............C.........................................................................................A..................................................................... B14**

**...................A.....T..............C.........................................................................................A..................................................................... B19**

**............................G..C..................A.............CA..C..........T...........T.....C..C.....T..AC.G.................C......................................T.............................. B22**

**...............................C..................A.............CA..C..........T...........T.....C..C.....T..A..G.................C......................................T.............................. D1(88)**

**~~~~~~~~1410~~~~~~1420~~~~~~1430~~~~~~1440~~~~~~1450~~~~~~1460~~~~~~1470~~~~~~1480~~~~~~1490~~~~~~1500~~~~~~1510~~~~~~1520~~~~~~1530~~~~~~1540~~~~~~1550~~~~~~1560~~~~~~1570~~~~~~1580~~~~~~1590~~~~~~1600~~~~~~**

**....|....|....|....|....|....|....|....|....|....|....|....|....|....|....|....|....|....|....|....|....|....|....|....|....|....|....|....|....|....|....|....|....|....|....|....|....|....|....|....|**

**GACCCGTCTCGGGGCCGTTTGGGNCTCTACCGTCCCCTTCTTCNTCTGCCGTTCCGGCCGACCACGGGGCGCACCTCTCTTTACGCGGTCTCCCCGTCTGTGCCTTCTCATCTGCCGGACCGTGTGCACTTCGCTTCACCTCTGCACGTCGCATGGAGACCACCGTGAACGCCCACCAGGTCTTGCCCAAGGTCTTACAT C2(242)**

**............................................C.A..............................................................................................................................A.......................... B10**

**.....T...........C..........CT...........C...........T..A...............................A....................................................................................A.......................... B6**

**..............................................A......................................................................A..........................................................................C....... B15**

**...........................................................A............................................................................................................G............................... B1**

**...........................................................V............................................................................................................................................ CD1(31)**

**...........................................................A....T....................................................................................................................................... B11**

**...........................................................A............................................................................................................................................ B20**

**...........................................................A..........................................................................................................A................................. B12**

**..............................................A..............................................................................................................................A.......................... B2**

**.....T...........C..........CT...........C...........T..A...............................A.........................................................................................AT.................... B7**

**..............................................A..............................................................................................................................A.......................... B14**

**..............................................A..............................................................................................................................A.......................T.. B19**

**.....T...........C..........CT...........C...........T..A...............................A.........................................................................................ATG................... B22**

**.....T...........C..........CT...........C...........T..A...............................A.........................................................................................AT.................... D1(88)**

**~~~~~~~~1610~~~~~~1620~~~~~~1630~~~~~~1640~~~~~~1650~~~~~~1660~~~~~~1670~~~~~~1680~~~~~~1690~~~~~~1700~~~~~~1710~~~~~~1720~~~~~~1730~~~~~~1740~~~~~~1750~~~~~~1760**

**....|....|....|....|....|....|....|....|....|....|....|....|....|....|....|....|....|....|....|....|....|....|....|....|....|....|....|....|....|....|....|....|...**

**AAGAGGACTCTTGGACTCTCAGCAATGTCAACGACCGACCTTGAGGCATACTTCAAAGACTGTKTGTTTAARGACTGGGAGGAGTTGGGGGAGGAGATTAGGTTAADGRTCTTTGTACTAGGAGGCTGTAGGCATAAATTGGTCTGTTCACCAGCACCATGCA C2(242)**

**....................G....C..........................................................C.............................................................................. B10**

**.................................................................................................................................................................A. B6**

**...........G..............................................................A..........................A........G......T.....................A...G..CG............... B15**

**..................................................................................................................................................C................ B1**

**............................................................................................................D...................................................... CD1(31)**

**................................................................................................C....A........G......T.........................G..CG..........-.... B11**

**.....................................................................................................................T............................................. B20**

**......................TC...................................................................C.........A........G......T.........................G..CG............... B12**

**...................................................................................................................................A............................... B2**

**......................TC........................................................................T....A...C..T.G......T.........................G..CG............... B7**

**......................................T..................................................................................................................-......... B14**

**................................................................................................................................................................... B19**

**......................TC............................................C................C..........C....A........G......T.........................G..CG............... B22**

**....................T.T..............................................................................A......N........T............................CG............... D1(88)**
